# Is there an omission effect in prosocial behavior? A laboratory experiment on passive vs. active generosity

**DOI:** 10.1371/journal.pone.0172496

**Published:** 2017-03-01

**Authors:** Manja Gärtner, Anna Sandberg

**Affiliations:** 1 Division of Economics, Department of Management and Engineering, Linköping University, Linköping, Sweden; 2 Institute for International Economic Studies (IIES), Stockholm University, Stockholm, Sweden; Technion Israel Institute of Technology, ISRAEL

## Abstract

We investigate whether individuals are more prone to act selfishly if they can passively allow for an outcome to be implemented (omission) rather than having to make an active choice (commission). In most settings, active and passive choice alternatives differ in terms of factors such as the presence of a suggested option, costs of taking an action, and awareness. We isolate the omission effect from confounding factors in three experiments, and find no evidence that the distinction between active and passive choices has an independent effect on the propensity to implement selfish outcomes. This suggests that increased selfishness through omission, as observed in various economic choice situations, is driven by other factors than a preference for selfish omissions.

## Introduction

Are people more selfish if they can passively allow for a selfish allocation of resources, rather than actively having to implement it? This question speaks to a variety of different choice situations, as decisions about resource allocations often vary by whether they require active involvement or not. People may be more likely to walk by a charity solicitor on the street than to refuse to give to a solicitor who knocks on their door. An employer may passively allow for inflation to erode workers’ real wages even if she would not be willing to actively cut nominal wages. Citizens face governmental policies about redistributive matters that vary by the involvement they require, such as whether being an organ donor requires active registration or not, or whether declarations of taxable income require more or less active statements. In this paper, we conduct three experiments to test whether individuals exhibit preferences for selfish omissions over selfish commissions in economic decision-making.

We define a choice as active, or as a *commission*, if the decision maker implements an outcome by taking an action, whereas we define a choice as passive, or as an *omission*, if the decision maker allows for an outcome to be implemented by being inactive. A body of research in moral philosophy and psychology suggests that individuals favor harmful omissions over harmful acts. Subjects faced with hypothetical choice scenarios often judge harmful acts as morally worse than equally harmful omissions [1–5] and report to be more willing to do harm passively rather than actively [6–11]. In this literature, the general tendency to favor harmful omissions over harmful commissions has been termed *omission bias*. In the current study, we are interested in the “pure” effect of passive choices, free of confounding factors such as suggestiveness, action costs and awareness. Throughout the paper we will call this the *omission effect*.

While research in other disciplines and evidence from real-world examples suggest that passivity matters, it has not been systematically investigated whether the distinction between commissions and omissions *itself* affects economic decision-making. In most choice situations, it is, in fact, impossible to identify the effect of such a distinction, since active and passive choices differ systematically across other dimensions.

Most importantly, it can be difficult to empirically separate the omission effect from the status quo effect (i.e. the effect of an alternative having been implemented previously) and the default effect (i.e. the effect of presenting an alternative as the suggested option). However, since status quo and default options can be implemented either passively or actively, the omission effect differs conceptually from the status quo and the default effect. A default option is sometimes referred to as the outcome that will prevail if a decision maker is passive. We use a broader definition, recognizing that situations may differ in the degree of activity required to confirm the choice of a suggested option. Thus, we distinguish between “passive defaults” (implementing the suggested option requires no activity whatsoever) and “active defaults” (implementing the suggested option requires some activity). For example, public policies such as organ donation regulations often involve defaults that can be accepted passively, while market settings such as online purchases and acceptance of user agreements often require active verification of a pre-selected default option (e.g. regarding user conditions, shipping, or receiving future emails from a retailer). Such active verification can for instance consist of pressing an “I agree”-button on a computer screen or signing a piece of paper. Previous research on the default effect shows that individuals are more likely to choose an alternative if it is presented as the default option (e.g. [12–15]). To determine the relevance of the distinction between commissions and omissions, it is crucial to disentangle the omission effect from the effect of introducing a suggested option. In order to test whether there exists an omission effect beyond the effect of a default option, we compare the effect of introducing an active default to the effect of introducing a passive default. The results of such an investigation should be of direct relevance for mechanism designers since if individuals react differently to active than to passive defaults, it might be possible to nudge behavior simply by changing whether a given default option needs to be actively confirmed or not.

Apart from the presence of a suggested option, commissions and omissions often differ in terms of the costs of taking an action and the decision maker’s awareness. In many choice situations remaining passive is less costly than taking an action. Making an active choice may require more costs in terms of time and effort, such as filling out an organ donation form. It may also be cognitively more costly for individuals that are unsure about what to choose to resolve this uncertainty under active choices. In some settings, passive choices might be the result of unawareness of being in a choice situation or a lack of enough time to make an active choice, rather than the expression of a preference. In such settings, self-serving commissions may be judged more harshly than self-serving omissions, merely due to differences in the revealed intent. For example, one motivation for legally distinguishing between the act of killing and the failure to help someone who is dying is that the former provides stronger evidence of harmful intentions. Our contribution is to provide a test that isolates the effect of active and passive choices from these other confounding factors (i.e., suggested options, costs of taking an action, and awareness). The main question we ask is whether there is an omission effect in the sense that individuals have a preference for implementing selfish options passively rather than actively.

We hypothesized that there is an omission effect in economic decision making above and beyond these confounding factors. There are several reasons for this. First, individuals may perceive the distinction between selfish commissions and selfish omissions as *morally significant in and of itself*. Accordingly, individuals that inherently care about behaving morally may reflect this distinction in their behavior. Such a moral distinction is not necessarily made consciously. Research by Cushman et al. [5], using fMRI data, indicates that omission effects tend to arise automatically, without the application of controlled cognition. In line with this, Sunstein [16] suggests that the act-omission distinction may operate as a “moral heuristic”. The idea is that, while the moral intuition of distinguishing between acts and omissions makes sense in most everyday situations, this principle is sometimes overgeneralized to situations where there is no morally relevant distinction between acts and omissions. Second, the distinction between selfish commissions and selfish omissions may be driven by *social esteem or self-image concerns*. It has been suggested that individuals care about their social- and/or self-image and are motivated by a concern for the value of reputation that is attached to different choices [17–20]. Such image concerns may be related to the outcomes of choices as well as to how these outcomes come about. Omissions are less salient than actions, making them less likely to be observed by others and more likely to be forgotten or repressed by the agent herself. Moreover, selfish omissions can appear less intentional than selfish actions, making them less likely to be judged negatively by oneself or others. Therefore, omissions may send weaker signals than commissions about an agent’s type, resulting in less stigma being associated with passive, as compared to active, norm violations.

These mechanisms suggest that the moral appropriateness of a choice may not just depend on the alternatives initially available to the individual and the selected outcome, but also on how an outcome comes about. This means that actions *themselves* may affect social norms and moral concerns. In line with this notion, Levitt and List [21] and Krupka and Weber [22] argue that a utility maximization framework that aims at explaining choices under social norms can benefit from including actions as an argument in the utility function. Whether an outcome follows from an active choice or from the decision maker merely allowing for the outcome to be implemented is one such difference in how outcomes come about. Accordingly, the question of whether the distinction between active and passive choices is an expression of preferences also relates to the broader issue of whether individual utility maximization can be modeled solely as a function of initial states and final outcomes.

We present three experiments aimed at isolating the distinction between omissions and commissions from confounding factors. In these experiments, we conduct a series of dictator games where subjects can choose between two different allocations of money between themselves and another participant: one selfish allocation and one fair allocation. In each game, one allocation is indicated by a pre-ticked box. We will refer to this allocation as the default option. Our treatments vary the relative stakes of the default, and whether the default allocation can be implemented by commission or by omission. We hypothesized that subjects facing a selfish default option, which implies violating a fairness norm, would be more likely to choose the default option by omission rather than by commission. Further, we hypothesized that the omission effect would be smaller for choices with a non-selfish, norm-compliant default option. However, our results show no statistically significant omission effect in the share of selfish choices, neither given a selfish nor given a non-selfish default option. Thus, we find no support for the hypothesis that there is an omission effect in prosocial behavior beyond the default effect and other confounding factors. All else equal, individuals do not prefer to implement a selfish default option passively rather than actively. We can show that this finding holds across a number of different allocation trade-offs with various properties as well as across settings with weak and strong default effects. Our result suggests that social preferences are not sensitive to an omission effect. Thus, increased selfishness through omission, as observed in various settings, is likely to be driven by other factors than a preference for selfish omissions. In particular, the presence of a suggested option, costs of taking an action and limited awareness are confounding factors which differ systematically across commissions and omissions in many settings and which may explain why passivity and selfish behavior often coincide. Each of these factors is discussed in the conclusion.

The remainder of the paper is organized as follows. Sections 2, 3 and 4 present the design and results of the first, second and third experiment. Section 5 concludes with a discussion of the implication of our results.

## Experiment 1

### Experimental design

The first experiment employs a repeated dictator game with 14 different binary allocation choices. In the first choice, all subjects face the same allocation trade-off between an allocation which is payoff-dominant for the dictator (the “*selfish”* allocation), giving 90 DKK (≈ 13.8 USD) to the dictator and 10 DKK (≈ 1.5 USD) to the recipient, and an allocation which is both fair and efficient (the “*fair”* allocation), giving 70 DKK (≈ 10.7 USD) each to the dictator and the recipient. Having an identical first choice across subjects allows for a between-subject analysis. The subsequent 13 choices have varying allocation trade-offs and follow in an order that is randomized at the individual level. These trade-offs differ in terms of the cost of giving and the size and direction of the payoff difference between the dictator and the recipient. Table A in S1 File lists all allocation trade-offs and their characteristics. For each new allocation choice, each subject is randomly rematched with an anonymous recipient in the same room. After the experiment, one choice is randomly chosen for payment.

Subjects are randomized into either the *commission treatment* or the *omission treatment* and remain within one treatment throughout the experiment. In both treatments subjects have 40 seconds to make each allocation choice, as indicated by a timer on the screen. For each choice, we randomly select one of the two allocations to be presented as the default, i.e. subjects either face a *selfish default* or a *fair default*. The default is indicated by a pre-ticked rather than an empty box beside that allocation. The difference between the commission treatment and the omission treatment is whether implementing the pre-ticked default allocation requires an active or a passive choice. To choose the default allocation, subjects in the commission treatment must actively press a confirm button that restates the allocation choice, while subjects in the omission treatment simply let the timer run down. In order to implement the alternative option, subjects in both treatments must tick the box beside that option and press another confirm button. After a choice has been made, subjects in both treatments must wait for the timer to run down in order to proceed to the next stage. We display screenshots of the decision interface in S1 and S2 Figs.

If a subject in the commission treatment fails to make an active choice before the timer runs down, the subject proceeds to the next stage without receiving any earnings from that choice. Prior to the decision, subjects in the commission treatment are informed that they need to press a button to confirm their allocation choice, and that they are only paid for the tasks that they complete according to the instructions. Thus, it should be clear to participants in the commission treatment that they are not paid for the choices in which they remain passive. This implies that there are in fact three potential outcomes in the commission treatment: (90,10), (70,70) and (0,0). Since the outcome from remaining passive (0,0) is clearly dominated by both outcomes from taking an action (90,10 and 70,70), we do not believe that the additional option of remaining passive affects the behavior of subjects in the commission treatment.

To ensure that we give subjects enough time to make a choice, we conducted a pilot study to elicit response times. Subjects in the pilot take at most 22 seconds to make a choice in the commission treatment without any time constraint (*N* = 16, *mean* = 12.34, *s*.*d*. = 4.98). By giving participants in the experiment 40 seconds to make a choice, i.e. almost twice as much time, we rule out that time constraints drive passive choices in the omission treatment. Note that subjects are not likely to incorrectly perceive 40 seconds to be too little time, since they participate in practice rounds prior to the experiment, with 40 seconds to make a practice choice. However, introducing a period length of 40 seconds implies that subjects who choose the default in the omission treatment have to face their passive choice for rather a long time. In order to make the omission situation more natural, we therefore introduce a second task. Throughout the experiment and in each treatment, subjects can work on a slider task [23]. While the 14 allocation choices appear sequentially on the left-hand side of the screen at regular intervals, the slider task constantly appears on the right-hand side of the screen. We show a screenshot of the slider task in S3 Fig. In this task, subjects use their mouse to position sliders at a target location. The total number of sliders that can be solved is not restricted and for each correctly positioned slider, the subject earns 0.01 DKK (≈ 0.001 USD). The slider task is repetitive, tedious and pays very little as compared to the dictator games. Hence, it should not crowd out incentives for participating in the dictator games. Before the experiment, subjects play practice rounds and answer control questions about the decision situation and the slider task. After the experiment, subjects are asked to complete a questionnaire eliciting demographic characteristics. The written instructions are provided in S6 File.

Table 1 illustrates our 2x2 design (selfish/fair default x commission/omission treatment), using the first allocation trade-off between (90,10) and (70,70) as an example. All choices in the experiment are structured accordingly.

**Table 1:** Overview of treatments in Experiment 1: Choice between (90,10) and (70,70).

		Default Allocation	Implementation of Default
*I*	Commission Treatment	(90,10)	Active
*II*	Omission Treatment	(90,10)	Passive
*III*	Commission Treatment	(70,70)	Active
*IV*	Omission Treatment	(70,70)	Passive

Our main hypothesis is that subjects prefer to be selfish by omission rather than by commission. In terms of the first allocation trade-off, we expect that subjects facing the selfish default (90,10) are more likely to choose (90,10) over (70,70), if the default is implemented passively (omission treatment) rather than actively (commission treatment).

#### Hypothesis 1

Given a selfish default, the share of default choices is higher in the omission treatment than in the commission treatment:

(Selfish choices/II) > (Selfish choices/I).

If individuals are uncertain about which allocation to choose and it is psychologically costly to resolve this uncertainty, they might remain passive to avoid costly contemplation. If so, any omission effect that we observe for selfish defaults may express a *general* omission effect, irrespective of the characteristics of the omission option, rather than a preference for selfish omissions over selfish commissions. To address this concern, we also elicit omission effects when the default is fair. We hypothesize that there is an omission effect under selfish defaults beyond what can be explained by preference uncertainty and, hence, that the omission effect under selfish defaults is larger than the omission effect under fair defaults. In terms of the first choice, we hypothesize that the increase in the propensity to choose the (90,10) default by omission rather than commission is larger than the increase in the propensity to choose the (70,70) default by omission rather than commission.

#### Hypothesis 2

The omission effect is larger for choices with a selfish default than for choices with a fair default:

(Selfish choices/II)−(Selfish choices/I) > (Fair choices/IV)−(Fair choices/III).

Our experimental design is well suited to isolate the distinction between omissions and commissions since the treatment conditions only differ in terms of activity, while relevant aspects other than this distinction are held constant. Most importantly, subjects face the same choice alternatives and the same default condition across the commission and the omission treatment. Second, any potential omission effect is unlikely to be driven by differences in the time invested or effort costs since subjects are given the same fixed decision time across treatments and since making an active choice only requires clicking a button to confirm the choice. Third, we calibrated decision time such that any omission effect in choices cannot be driven by differences in time pressure across treatments. Finally, since subjects in both treatments have enough time to make an active choice and are fully informed about the structure of the choice situation before the experiment starts, there is little reason to believe that unawareness of the choice situation or lack of time will drive any omission effect in our setting. Holding these confounding factors constant, we therefore argue that any observed behavioral difference across treatments must be caused by the distinction between omissions and commissions *itself*.

### Ethics

According to guidelines from the Swedish research council, approval from an ethics committee is not required for behavioral studies that do not fall under the Swedish law concerning the Ethical Review of Research Involving Humans (SFS 2003:460). Since the experiments in our study do not include physical interventions or treatment of sensitive personal information (such as handling of social security numbers), this research project did not require review. For detailed information about the Swedish law, we refer the reader to our ethics statement in S2 File. All our studies (both in the laboratory and online) clearly indicated to the subjects that the study was conducted for the purposes of research and that the data would be analyzed anonymously. The collected data cannot in any way be connected to personal identifiers, such as social security numbers, and do not contain other sensitive personal information. All subjects participated voluntarily in the studies and were at least 18 years old. Participants in the laboratory experiment described in the current section had voluntarily signed up to participate in the subject pool at the Centre for Experimental Economics (http://www.econ.ku.dk/cee/participate/), and thus been informed of the rules that the laboratory applies. Similarly, participants in the online experiments described in subsequent sections had voluntarily registered as workers at the online labor market and then actively chosen to participate in the study. All studies were conducted under full-disclosure, meaning that there was no deceit. All treatments were non-invasive and subject payments were made as indicated to subjects in the study.

For these reasons, we did not ask the Central Ethical Review Board (Etikprövningsnämnden) for a written waiver or collected explicit written informed consent from the subjects. There has been no such praxis present in our field of study in Sweden. The decision in Sweden is and has been the discretion of the individual researcher and research departments. The Department of Economics at Stockholm University and the Department of Economics at the Stockholm School of Economics (Sandberg's employer at the time of the study) did not consider the study being eligible for review or that it required collecting informed consent in line with the Swedish legislation on ethical review.

### Results

400 subjects were recruited from the subject pool of the University of Copenhagen, using the online system ORSEE [24]. The experiment took place at the laboratory at the local Center for Experimental Economics (CEE) in May 2013 and the experimental software z-Tree [25] was used. In total we ran 15 sessions, each session lasting roughly one hour. Subjects earned about 141 DKK (≈ 21.6 USD) on average. At most, a subject solved 327 sliders in total, resulting in a payment of 3.27 DKK from the slider task. There is no significant difference between the commission treatment and the omission treatment in the total number of sliders solved (162.7 vs. 162.8, *t* = -0.019, *p* = 0.985), the propensity to solve at least one slider in total (98.5% vs. 99%, *χ*^*2*^*(1)* = 0.203, *p* = 0.653), or the propensity to solve at least one slider in every round (68.5% vs. 64%, *χ*^*2*^*(1)* = 0.906, *p* = 0.341).

The average participant age is 26. 48% of the participants are women, 26% are Danish, and 78% are full-time students. We show participant characteristics in Table B in S1 File. 200 subjects participated in the commission treatment and 200 in the omission treatment. Participants in the commission treatment largely managed to make an active choice by clicking the confirm button within the allotted time. In total, participants in the commission treatment failed to make an active choice in 2.6% of all choice occasions (72 out of 2800 choices). Looking only at the first choice, the equivalent number is 3% (6 out of 200 choices). We exclude observations for which this was not the case from the main analysis, but our main results are robust to including them under various assumptions (we present these robustness checks in S5 File). Below, we first present the results from the first allocation choice, and then from all 14 allocation choices pooled.

#### First allocation choice

**Fig 1:**
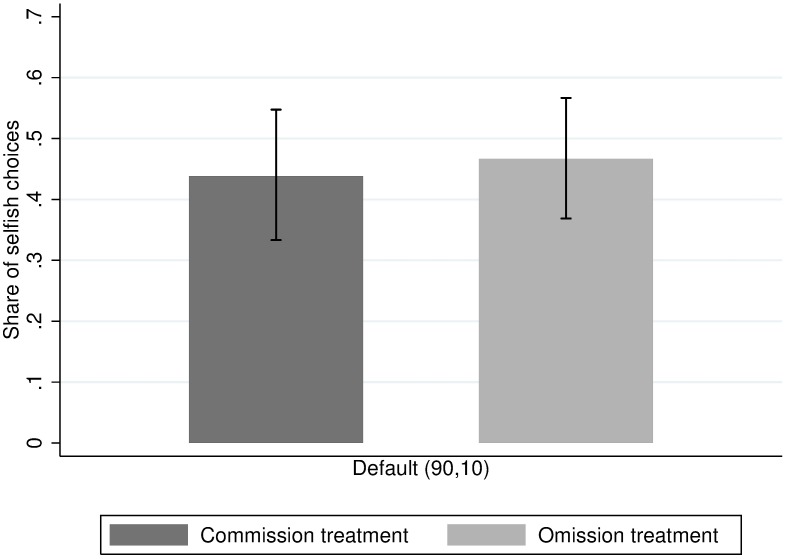
Average share of selfish choices in the first allocation choice with default (90,10).

In the first choice, the subject can either allocate 90 DKK to herself and 10 DKK to the recipient, or 70 DKK each to herself and the recipient. Overall, the share of selfish choices is 43.4%, which is within the range found in previous studies using binary dictator games with a similar structure. As shown in Fig 1, when the selfish allocation (90,10) is presented as the default option, it is chosen by 43.8% of the subjects in the commission treatment and 46.7% of the subjects in the omission treatment. We cannot reject the null hypothesis that the probability of choosing the selfish option is the same across these two conditions (*χ*^*2*^*(1)* = 0.16, *p* = 0.691). Thus, we do not find support for our hypothesis that subjects are more likely to choose a selfish default in the omission treatment than in the commission treatment.

We also run an OLS regression, using an indicator for whether the participant chose the (90,10) default option as the dependent variable (*β* = 0.028, *s*.*e*. = 0.072). The 95% confidence interval of the estimated omission effect is [-0.114,0.171]. Thus, while the point estimate is very small and not nearly statistically significant, we can only rule out the existence of an effect larger than 17.1 percentage points.

The share of selfish choices, across treatments and for both selfish and fair defaults, is presented in Table 2. Since we find no evidence for an omission effect in selfish choices, there are no mechanisms to distinguish. Therefore, we leave the details of the analysis of the fair default and our second hypothesis to S3 File. In short, when the fair allocation (70,70) is presented as the default option, there is no statistically significant difference in the share of fair choices between the two treatments. Moreover, testing our second hypothesis, we cannot reject the null-hypothesis of no difference in omission effects between the fair and the selfish default conditions.

**Table 2:** Share of participants choosing the selfish allocation in the first choice.

	Commission treatment	Omission treatment	Total
Default (90,10)	43.8%	46.7%	45.4%
	(*n* = 89)	(*n* = 105)	(*n* = 194)
Default (70,70)	36.2%	47.4%	41.5%
	(*n* = 105)	(*n* = 95)	(*n* = 200)
Total	39.7%	47.0%	43.4%
	(*n* = 194)	(*n* = 200)	(*n* = 394)

*Note*: In each cell we show the share of participants choosing the selfish allocation (90,10). In parentheses we show the total number of participants in each cell.

#### All allocation choices

We also test our hypotheses using data from all 14 allocation choices. Table C in S1 File tests our first hypothesis for each choice separately. The difference in default choices between the omission and the commission treatment is significant at the 5 percent level for only one of the 14 choices. When correcting the significance level for multiple testing (a Bonferroni correction with *n* = 14 and *α* = 0.05 yields an adjusted required significance level of 0.05/14≈0.004), no individual treatment effect is statistically significant. Since we do not find any order effects in the likelihood to choose the default, in the analyses below we pool all choices.

By pooling all choices we can substantially increase the precision of our estimates, compared to when restricting the data to the first allocation choice. To test our first hypothesis, we look at all choices with a selfish default, i.e. all allocation trade-offs where the default option is strictly payoff-dominant for the dictator. Table 3 shows the results from OLS regressions, using an indicator for whether the participant chose the selfish default allocation, or not, as the dependent variable. The omission effect is given by the coefficient of the explanatory variable *Omission treatment*. Model 1 restricts the sample to only the first choice, and verifies the result from the previous section (*β* = 0.028, *p* = 0.69). Model 2 pools all allocation choices with a selfish default, i.e. all allocation trade-offs where the default option is strictly payoff-dominant for the dictator. There is no significant omission effect in the likelihood to choose a selfish default even when pooling all choices (*β* = 0.004, *p* = 0.90), and the 95% confidence interval narrows from [-0.114,0.171] in Model 1 to [-0.056,0.064] in Model 2.

**Table 3:** Treatment effect on the propensity to choose the selfish default.

	Model 1	Model 2	Model 3	Model 4	Model 5
Omission treatment	0.028	0.004	0.046	-0.019	0.020
	(0.072)	(0.030)	(0.041)	(0.030)	(0.039)
Constant	0.438***	0.666***	0.515***	0.758***	0.554***
		(0.020)	(0.029)	(0.020)	(0.028)
*R*^*2*^	0.00	0.00	0.00	0.00	0.00
*N* (choices)	194	2,576	1,347	1,227	998
Choices included:	First choice	Selfish vs. non-selfish	Selfish vs. non-selfish (not behind)	Selfish vs. behind	Selfish vs. efficient

*Note*: OLS regressions. The sample is restricted to choices where the default option is selfish (strictly payoff dominant for the dictator). Dependent variable: = 1 if default chosen, = 0 otherwise. *Model 1* only includes the first choice between (90,10) and (70,70). *Model 2* includes all allocation choices except choice number 13 which has no strictly payoff dominant option for the dictator. *Model 3* includes choices 1, 2, 3, 4, 5, 6 and 10. *Model 4* includes choices 7, 8, 9, 11, 12 and 13. *Model 5* includes choices 1, 6, 9, 10 and 12. See Table A in S1 File for a list of all choices. Standard errors are clustered on participant in all models except *Model 1*.

*** *p*<0.01 (the absence of asterisks indicates p>0.10).

Next, to see whether the omission effect varies across allocation trade-offs with different properties, we split our data into different subsets of choices. Model 3 restricts the analysis to allocations where the dictator’s payoff is at least as high as the recipient’s payoff. Model 4 only includes choices that trade-off selfishness and behindness, i.e. choices between one option which is weakly payoff-dominant for the dictator and another option giving the dictator less than the recipient. Model 5 is restricted to the choices where the selfish option is less efficient than the non-selfish option. Also for these subsets of choices, there is no significant omission effect. The 95% confidence intervals for the estimated coefficients are [-0.035,0.127], [-0.079,0.040] and [-0.079,0.040], respectively. Hence, even when substantially increasing the precision of the estimated omission effect by pooling all choices, we find no evidence that individuals are more likely to be selfish passively rather than actively. As in the previous section, we leave the analysis of the fair default to S3 File.

In S4 File we also test our first hypothesis by estimating a latent class model, classifying subjects into different social preference types. This allows us to take inequity aversion and social welfare preferences into account in the same model. In line with our previous results, we find no significant difference in the distribution of types between the omission and the commission treatment under selfish default options.

#### Default effects

To evaluate the size of the default effect in Experiment 1, we compare the share of selfish choices when the selfish allocation is the default to the share of selfish choices when the fair allocation is the default. In the first allocation choice, the share of (90,10) choices is not significantly larger when (90,10) is the default as compared to when (70,70) is the default, neither in the commission treatment (43.8% vs. 36.2%, *χ*^*2*^*(1)* = 1.17, *p* = 0.28), nor in the omission treatment (46.7% vs. 47.4%, *χ*^*2*^*(1)* = 0.01, *p* = 0.92).

We estimate default effects for the pooled choices by running separate regressions for each treatment, using an indicator variable for selfish choice as the dependent variable, and an indicator variable for selfish default as the explanatory variable. Using the sample restriction from Model 2 in Table 3, there is a small but significant default effect in the commission treatment (*β* = 0.053, *p* = 0.01) but not in the omission treatment (*β* = 0.07, *p* = 0.80). Using the sample restrictions from Models 3–5, there are no significant default effects, neither in the commission treatment nor in the omission treatment. Assuming that the default effects for selfish and fair defaults point in the same direction, this means that we find no strong evidence of default effects in Experiment 1.

## Experiment 2

In Experiment 1, we find no evidence of an omission effect on prosocial behavior. However, nor do we find any strong evidence of a default effect. Thus, from Experiment 1, we can conclude that there is no evidence of an omission effect independent of the default effect. However, given that default and omission effects have been suggested to be based on similar mechanisms (see e.g. [26]), it cannot be ruled out that an omission effect only occurs if these mechanisms are clearly at work, i.e. in the presence of a positive default effect. To test for the existence of an omission effect in the presence of a positive default effect, Experiment 2 introduces a setting where we expect the default effect to be stronger. For this purpose, we present the default as an entitlement, rather than introducing it as a randomly selected pre-ticked box.

### Experimental design

The second experiment employs a one-shot dictator game with one binary allocation choice. The relative payoff between the two allocations resembles that used in the first choice in Experiment 1. Subjects choose between an allocation which is payoff-dominant for the dictator (the *selfish* allocation), giving $1.05 to the dictator and $0.05 to the recipient, and an allocation which is both fair and efficient (the *fair* allocation), giving $0.70 each to the dictator and the recipient. Each subject is randomly assigned to one of three treatments: the commission treatment, the omission treatment or the no-default treatment. After the experiment, subjects are randomly matched and randomly assigned to either the role of the dictator or the role of the recipient and paid accordingly. As in Experiment 1, subjects have 40 seconds to make their choice, as indicated by a timer on the screen. To simplify the procedure, we do not include the slider task that was used in Experiment 1.

In the *commission treatment* and the *omission treatment*, the selfish allocation is presented as the default. To create a stronger default effect than in Experiment 1, we present the default allocation as an initial distribution rather than a pre-ticked box. Before making the choice, participants in these two treatments read instructions stating that “You and the other worker receive the following bonus payments for this task: You: $1.05, Other: $0.05”. Participants are then informed that they can either confirm this bonus payment, or select an alternative option. Subjects in both treatments can actively choose either the selfish default allocation or the alternative, fair allocation by ticking an empty box next to the allocation. In the omission treatment, subjects can also implement the selfish allocation by doing nothing and letting the timer run down. Thus, as in Experiment 1, the difference in the share of selfish choices between the commission treatment and the omission treatment measures the omission effect. Note that the only difference between the commission treatment and the omission treatment is that subjects in the omission treatment have the *additional* option of implementing the selfish allocation by remaining passive.

We introduce the *no-default treatment* to measure the size of the default effect. The no-default treatment has no default option, but equals the commission treatment in all other respects. The difference in the share of selfish choices between the no-default treatment and the commission treatment allows us to measure the default effect. Table 4 illustrates the differences between treatments. The experimental instructions can be found in S7 File.

**Table 4:** Overview of treatments in Experiment 2: Choice between (1.05,0.05) and (0.70,0.70).

	Default Allocation	Implementation of Default
Commission Treatment	(1.05,0.05)	Active
Omission Treatment	(1.05,0.05)	Passive or Active
No-default Treatment	No Default	-

We hypothesize that there is a default effect in this setting, i.e. that subjects are more likely to choose the selfish allocation in the commission treatment than in the no-default treatment. If there is an omission effect, subjects will be more likely to choose the selfish default in the omission treatment than in the commission treatment. If there is no omission effect, even in the presence of a default effect, there will be no difference between the commission and the omission treatment.

### Results

453 subjects were recruited through a work task posted on Amazon Mechanical Turk in August 2014. Subjects received a fixed payment of $0.50 for participation on top of their earnings from the experiment, which were paid as bonuses. To avoid non-random attrition from treatments and to select only sufficiently motivated subjects, the experiment started with a short transcription task that asked subjects to transcribe a text from a picture into a text box. The experiment was part of a larger study, but the dictator game choices reported here were elicited first. After the dictator game, subjects participated in an additional task and answered a short questionnaire.

**Fig 2:**
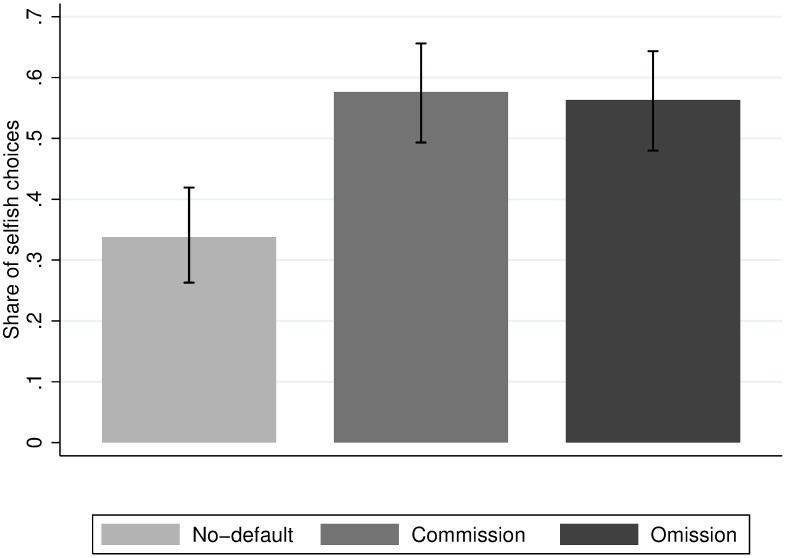
Average share of selfish choices across treatments.

40% of the participants are women and 75% are located in the US. 151 subjects participated in each treatment. No subject in the commission treatment and only 2 subjects (1.3% of subjects) in the no-default treatment failed to make a choice in the allotted time. These shares are not significantly different across the two treatments (*χ*^*2*^*(1) =* 2.01, *p* = 0.16). We include these two subjects in the analysis presented below, but our results are robust to excluding them. Table 5 presents the share of subjects choosing the selfish option across treatment conditions. Fig 2 illustrates the findings.

**Table 5:** Share of participants choosing the selfish allocation in Experiment 2.

	No-default treatment	Commission treatment	Omission treatment	Total
Default (1.05,0.05)	33.8%	57.6%	56.3%	49.2%
	(*n* = 151)	(*n* = 151)	(*n* = 151)	(*n* = 453)

*Note*: In each cell we show the share of participants choosing the selfish allocation (1.05,0.05). In parentheses we show the total number of participants in each cell.

First, we test whether we succeeded in introducing a default effect into the decision. In the no-default treatment, 33.8% of the subjects choose the selfish option. Presenting the selfish option as the default in the commission treatment significantly increases the share of selfish choices by 23.8 percentage points (*χ*^*2*^*(1) =* 17.29, *p* = 0.00). This corresponds to an increase in selfish choices of 70.4% through the introduction of a selfish default. Thus, as hypothesized, subjects are more prone to choose the selfish option when it is presented as the default.

Second, we test whether an omission effect occurs in the presence of this default effect. The difference in the share of selfish choices in the commission treatment and the omission treatment is small with 1.3 percentage points and statistically insignificant (*χ*^*2*^*(1) =* 0.054, *p* = 0.816). Thus, even in a context where the default effect is strong and the mechanisms behind a potential omission effect are at work, we find no evidence that the possibility of choosing a selfish default passively rather than actively increases the share of selfish choices. The 95% confidence interval of the estimated omission effect is [-0.126,0.099]. Hence, assuming that the effect is positive, we can rule out the existence of an omission effect of 10 percentage points or larger.

Subjects in the omission treatment could implement the selfish default allocation either actively (by ticking the box) or passively (by letting the timer run down). Among the subjects that choose the selfish option in the omission treatment, 10.6% use the possibility to remain passive. This corresponds to 6% of all subjects in the omission treatment, which is a significantly larger share than the subjects failing to make an active choice in the no-default treatment and the commission treatment (omission vs. commission: *χ*^*2*^*(1) =* 9.30, *p* = 0.00; omission vs. no-default: *χ*^*2*^*(1) =* 4.62, *p* = 0.03). Thus, a significant, but small, fraction of subjects in the omission treatment consciously use the option to be selfish by omission. However, since the share of selfish choices is the same across the omission and the commission treatments, choosing the selfish option passively only seems to serve as a substitute to being selfish by commission.

## Experiment 3

So far, we found no evidence of an omission effect, even in the presence of a substantial default effect. However, one remaining concern may be that, in Experiments 1 and 2, subjects who make a passive choice must wait 40 seconds to let the timer run down in order to confirm their choice. Facing the choice situation for such a long time may cause psychological discomfort for subjects intending to choose the selfish option. If such psychological discomfort counteracts the omission effect, reducing the time that subjects have to make their choice should lead to an increase in the omission effect. Thus, in Experiment 3, we test if our results are robust to a shorter decision time. We thank an anonymous reviewer for this suggestion.

### Experimental design

In the third experiment we reduce the decision time from 40 seconds to 20 seconds. Our pilot study, as reported in Experiment 1, showed that subjects take at most 22 seconds to make a choice in the commission treatment without any time constraint. Thus, rounding to the nearest ten, we argue that 20 seconds should allow subjects to make a choice before the timer runs down in all treatments. This should also be enough time for subjects in all treatments to make an active decisions, and to prevent subjects from using shortage of time as a credible excuse for being selfish [27]. In all other respects, the design of Experiment 3 is identical to that of Experiment 2.

To assess how the reduced decision time affects the time pressure and stress experienced by subjects, we also replicate two questions from the questionnaire of Experiment 2. Subjects are asked to rate (i) whether the time was sufficient for making a choice on a scale from 1 (“strongly disagree”) to 7 (“strongly agree”), and (ii) how pressured they felt by the time constraint on a scale from 1 (“not at all time pressured”) to 5 (“very time pressured”).

### Results

We recruited 448 subjects through a work task posted on Amazon Mechanical Turk in November 2016. Subjects received a fixed payment of $1.00 for participation on top of their earnings from the experiment, which were paid as bonuses. 45% of the participants are women and all participants are located in the US. No subject in the no-default treatment and only one subject (0.67%) in the commission treatment failed to make a choice within the allotted time. The share of subjects failing to make an active choice is not significantly different between these two treatments *χ*^*2*^*(1)* = 1.00, *p* = 0.32). We include this subject in the analysis presented below, but our results are robust to excluding the subject.

Compared to subjects in Experiment 2, subjects in this experiment agree less with the statement that they had sufficient time to make their choice. This different is significant but fairly small (6.51 vs. 6.65, *z* = 2.57, *p* = 0.01). Importantly, 95.1% of subjects agree that time was sufficient. Similarly, subjects report significantly stronger feelings of time pressure in this experiment than in Experiment 2 (1.77 vs. 1.49, *z* = -4.30, *p*<0.01), but 91.3% of subjects still report that they felt little or no time pressure. Thus, while the more restrictive time constraint significantly affected subjects’ perception of the available decision time, most subjects still agree that 20 seconds is sufficient time for making an informed choice without much time pressure.

**Fig 3:**
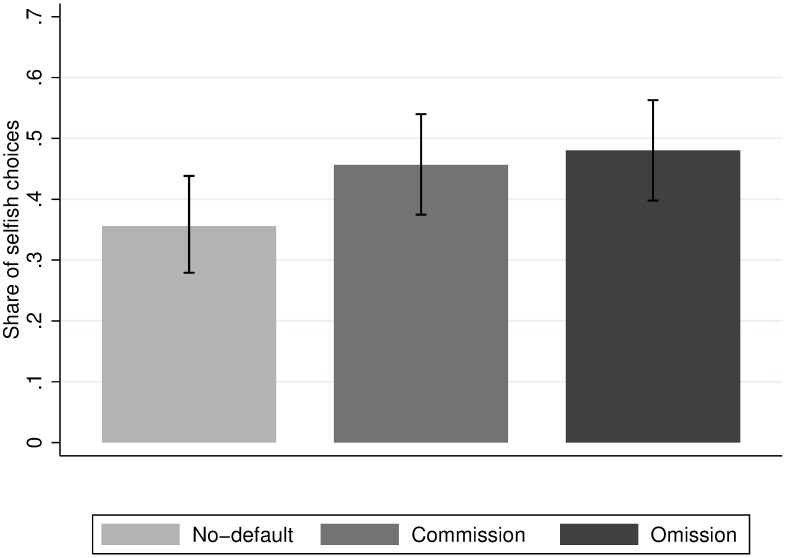
Average share of selfish choices across treatments in Experiment 3.

Table 6 presents the share of subjects choosing the selfish option across treatment conditions and Fig 3 illustrates these findings. Subjects are 10 percentage points more prone to choose the selfish option in the commission treatment than in the no-default treatment (*χ*^*2*^*(1) =* 3.13, *p* = 0.08). Thus, we find evidence of a default effect, albeit the size of this effect is 14 percentage points smaller than in Experiment 2 (*p* = 0.08 from a joint regression).

**Table 6:** Share of participants choosing the selfish allocation in Experiment 3.

	No-default treatment	Commission treatment	Omission treatment	Total
Default (1.05,0.05)	35.6%	45.6%	48.0%	43.1%
	(*n* = 149)	(*n* = 149)	(*n* = 150)	(*n* = 448)

*Note*: In each cell we show the share of participants choosing the selfish allocation (1.05,0.05). In parentheses we show the total number of participants in each cell.

The share of selfish choices in the omission treatment is only 4.4 percentage points larger than in the commission treatment. This difference is not statistically significant (*χ*^*2*^*(1) =* 0.17, *p* = 0.68). Thus, even when substantially reducing the time subjects need to wait when making a passive choice, we find no evidence that the possibility of choosing a selfish default passively rather than actively increases the share of selfish choices. The 95% confidence interval of the estimated omission effect is [-0.090,0.138], ruling out effect sizes above 13.8 percentage points.

As in Experiment 2, subjects in the omission treatment could implement the selfish default allocation either actively (by ticking the box) or passively (by letting the timer run down). Among the subjects who chose the selfish option in the omission treatment, 9.7% used the possibility to remain passive. In total, this corresponds to 4.7% of subjects in the omission treatment, which is a significantly larger share than the share of subjects remaining passive in the other treatments (omission vs. commission: *χ*^*2*^*(1) =* 4.58, *p* = 0.03; omission vs. no-default: *χ*^*2*^*(1) =* 7.12, *p* = 0.01). This suggests that, as in Experiment 2, a small but significant share of subjects in the omission treatment consciously use the option to be selfish by omission. However, again, the lack of difference in selfishness between the commission treatment and the omission treatment indicates that choosing the selfish option passively is merely a substitute to choosing the same option actively.

Taken together, we find no evidence that our findings are affected by reducing the decision time by half.

## General discussion

We find no support for the hypothesis that there is an omission effect in prosocial behavior beyond the default effect. All else equal, individuals do not prefer to implement a selfish default option passively rather than actively. This holds true both in a setting with weak default effects (Experiment 1) and a setting with stronger default effects (Experiments 2 and 3). This indicates that prosocial preferences do not depend on the difference between commissions and omissions as attributes of the action taken by a decision maker. It has been suggested that a utility maximization framework in the presence of social norms may benefit from including actions as an argument of the utility function (see, for example, [21,22]). Our results show that prosocial preferences are stable with respect to whether the choice is active or passive. This finding relates broadly to previous research on the sensitivity of dictator game behavior to contextual changes. Several studies show that seemingly small manipulations of the choice structure, such as relaxing the transparency of the one-to-one mapping between the dictator’s actions and the final outcomes [27] or varying the choice set [28,29], can have strong effects on levels of giving. But not all manipulations affect giving behavior. For instance, Dreber et al. [30] find no significant social framing effect when changing the name of the dictator game or the labeling of strategies. We complement previous research on giving in dictator games by providing a manipulation targeting the nature of the action taken by the dictator that has *no* effect on levels of giving.

Our result suggests that increased selfishness through omission, as observed in various settings, is not an omission effect in the sense that it expresses an individual’s preference for selfish omissions. Rather, observed behavioral differences between passive and active choices are likely to be driven by other factors than the difference between active and passive choices in and of itself. This leads to the question of which other situational factors could explain why passivity and selfish behavior often coincide. Below, we discuss the potential role of default and status quo effects, costs of taking an action, time constraints and awareness as potentially important confounding factors that commonly also differ across commissions and omissions in previous economics literature.

First, the omission option is a passive default option. Default effects have been identified in a variety of consequential real-world decisions, such as whether to become an organ donor [31,32], the choice of retirement savings plan [13], auto insurance [12] and energy provider [33]. Presenting a choice alternative as the default has also been found to affect dictator game giving, either through a self-serving interpretation of entitlements [15], or through resolving preference uncertainty [14]. Further, Grossman [34] finds that passive defaults can affect strategic ignorance in a dictator game. He lets dictators choose whether to remain ignorant about the payoff consequences of their choice for the recipient, thereby allowing them to choose a higher payoff for themselves without knowingly violating a fairness norm. The share of dictators choosing to remain ignorant increases from 25% when the choice is active and has no default, to 54% when the passive default option is to remain ignorant. However, since Grossman’s [34] study is restricted to comparing a setting with a passive default option to a setting with no default option, we cannot know if the observed effect is solely driven by the effect of introducing a suggested option, or if the possibility to passively implement the default plays an additional role. In fact, given the current state of evidence, it is generally hard to disentangle omission effects from default effects. While some default options are chosen passively, such as through setting a passive policy default or maintaining a contract, and others require active verification, such as through signing a document or renewing a contract, to our knowledge, no previous study systematically investigates the distinction between active and passive defaults. Our study provides a first test, indicating that, all else equal, individuals do not prefer passive over active defaults. Thus, it is likely that observed increases in selfish behavior in settings with passive default options are driven by the default effect rather than a preference for selfish omissions.

Second, commission and omission options can differ substantially in terms of action costs. In our experimental setting action costs are reduced to a minimum, since making an active choice only requires clicking a button. An example of a field setting with minimal action costs is online purchases where choosing to add options, such as environmentally friendly delivery or a charity donation, only requires checking a box. However, in some real life situations, active choices are associated with considerable investments of effort and time. For instance, actively choosing to become an organ donor may require both time and effort, such as filling out a form and reflecting on the ethicality of one’s choice. Similarly, making an informed decision about a new retirement plan requires a careful evaluation of all alternatives. Thus, in some settings, an increased propensity to stay passive may simply reflect the costs associated with making an active choice.

Third, it can be the case that passive choices, but not active choices, result from unawareness of the choice situation. In such settings, an increased willingness to be selfish by omission rather than commission can arise because agents are genuinely unaware of the possibility to commit a non-selfish act. Or, it can arise because they use this potential unawareness as an argument when justifying their choice to remain passive. For instance, individuals may be more likely to passively walk by a silent charity solicitor, allowing for potential unawareness, as compared to actively refraining from contributing if the solicitor explicitly asks for a contribution. Along these lines, several studies find that when given the possibility to opt-out of a situation in which a fairness norm would suggest a monetary contribution, some individuals choose this opt-out option [35–38]. For instance, Andreoni et al. [36] find that when placing a silent solicitor, rather than a solicitor who approaches customers, at one of two doors of a supermarket, the share of customers using this door increases from 35.6% to 52.8%. Our result suggests that an omission effect itself cannot explain this type of avoidance behavior. Rather, it is plausible that a silent solicitor allows for some ambiguity about whether a customer was actually aware of the presence of the solicitor, and that customers use this potential unawareness as an argument when justifying their choice not to contribute.

Fourth, time constraints may be used by agents as a credible excuse for being selfish by omission. Dana et al. [27] conduct a binary dictator game in which dictators can either make an active choice or passively let a timer cut them off at a random point within a 10 second interval, allowing for a random mechanism to make the choice. They find that 24 percent of the dictators passively allow for being cut off by the timer. Our finding indicates that, rather than being driven by an omission effect, the dictators in Dana et al. [27] are driven by other motivations. For instance, they may be using the shortage of time allowed for making an active choice as a credible excuse to remain passive.

Investigating the specific role of each of the above-mentioned mechanisms is beyond the reach of this paper. While our second and third experiments indicate that the presence of a default plays a non-negligible role, an interesting direction for future research would be to more systematically address the importance of costs of taking an action, plausible unawareness and time constraints in the same setting. As suggested by an anonymous reviewer, one natural extension to the current study would be to first replicate previous findings of an omission effect and then remove each of these confounding factors one at a time.

Furthermore, our experiment focuses specifically on omission effects in *prosocial behavior* in a context with *economic outcomes*. Whether omission effects exist for decisions that affect only the decision-maker, e.g. choices relating to risk or time preferences, is still an open empirical question. For example, consider the risky decision of whether to sell stocks at a loss or not. This decision may vary both in terms of the presence of a default (a specific price at which to sell may, or may not, have been suggested by the broker) and whether the default is implemented passively or actively (a stop-loss order to sell at a certain price may be implemented automatically, or require a call to the broker). Relatedly, Ritov and Baron [39] conduct a series of vignette studies, focusing on adverse outcomes from risky choices (e.g. investment decisions) that affect only the decision-maker. They find that individuals’ tendency to maintain the status quo is—at least partly—confounded with a bias in favor of remaining passive. An interesting question for future research is to see whether this finding holds up when keeping the suggested option constant across scenarios.

Similarly, we cannot rule out the possibility of an omission effect in prosocial choices with non-economic outcomes. Gärtner [40] suggests that the outcome-domain matters for moral decision making. In a vignette study on moral choices across different outcome domains, she finds that the omission effect in the so-called trolley dilemma is significantly smaller for decisions concerning loss of property than for decisions concerning physical harm. While this opens up for the possibility that our findings may be outcome-domain-specific, we leave these questions for future research.

Finally, while we find no omission effect in choices, it is possible that there is nevertheless an omission effect in the moral judgment of others’ choices. Previous vignette studies in psychology [1–5] suggest that observers judge harmful acts as morally worse than equally harmful omissions. Moreover, previous incentivized experiments on fairness that look at the punishment of unfair behavior find results relating to the omission effect. Coffman [41] studies third party punishment in a dictator game and finds that dictators who take money directly from the recipient are punished more than dictators who delegate the implementation of the same outcome to an intermediary with no other option than to implement the outcome. This suggests that third party judgment is affected by whether the action taken by the dictator is directly associated with the selfish outcome. DeScioli, Christner and Kurzban [42] also study third party punishment, conducting a dictator game in which a selfish but dominated allocation is indicated as the passive default option. They find that dictators choose this passive default more often when a punisher is present as compared to when there is no punisher. Cox et al. [43] study second party punishment in an ultimatum game, varying which of two allocations is the status quo option. They find that selfish choices are punished less often by the recipient when the selfish option is the status quo, as compared to when the fair option is the status quo. These studies suggest that when punishing the behavior of others, individuals take into account whether an unfair allocation is implemented as a passive default [42] and whether it is the initial endowment that needs to be implemented actively [43]. However, at least two important questions still need to be addressed. First, is there an omission effect in the punishment of selfish behavior, independent of the effect of defaults on punishment? Second, is there an omission effect in prosocial behavior, independent of the default effect, in the presence of punishment opportunities?

## Supporting information

S1 Fig Screen shot of omission treatment. (TIF)

S2 Fig Screen shot of commission treatment. (TIF)

S3 Fig Screen shot of slider task. (TIF)

S1 File Additional tables. (PDF)

S2 File Ethics statement. (PDF)

S3 File Results for fair default and second hypothesis. (PDF)

S4 File Structural estimation. (PDF)

S5 File Robustness checks. (PDF)

S6 File Instructions for Experiment 1. (PDF)

S7 File Instructions for Experiment 2. (PDF)

S1 Data Data from Experiment 1. (XLS)

S2 Data Data from Experiment 2. (XLS)

S3 Data Data from Experiment 3. (XLS)

S1 Code STATA code for Experiment 1. (TXT)

S2 Code STATA code for Experiment 2. (TXT)

S3 Code STATA code for Experiment 3. (TXT)

## References

[1] Spranca M, Minsk E, Baron J. Omission and commission in judgment and choice. Journal of Experimental Social Psychology 1991; 27(1): 76–105.

[2] Kordes-de Vaal JH. Intention and the omission bias: Omissions perceived as nondecisions. Acta Psychologica 1996; 93(1): 161–172. 8826794 10.1016/0001-6918(96)00027-3

[3] Cushman F, Young L, Hauser M. The role of conscious reasoning and intuition in moral judgment testing three principles of harm. Psychological Science 2006; 17(12): 1082–1089. 10.1111/j.1467-9280.2006.01834.x 17201791

[4] DeScioli P, Bruening R, Kurzban R. The omission effect in moral cognition: Toward a functional explanation. Evolution and Human Behavior 2011; 32(3): 204–215.

[5] Cushman F, Murray D, Gordon-McKeon S, Wharton S, Greene JD. Judgment before principle: engagement of the frontoparietal control network in condemning harms of omission. Social Cognitive and Affective Neuroscience 2011; 7(8): 888–895. 10.1093/scan/nsr072 22114079 PMC3501704

[6] Ritov I, Baron J. Reluctance to vaccinate: Omission bias and ambiguity. Journal of Behavioral Decision Making 1990; 3(4): 263–277.

[7] Baron J, Ritov I. Reference points and omission bias. Organizational Behavior and Human Decision Processes 1994; 59(3): 475–498. 10.1006/obhd.1999.2839 10433898

[8] Cohen BJ, Pauker SG. How do physicians weigh iatrogenic complications? Journal of General Internal Medicine 1994; 9(1): 20–23. 8133346 10.1007/BF02599137

[9] Asch DA, Baron J, Hershey JC, Kunreuther H, Meszaros J, Ritov I.,et al Omission bias and pertussis vaccination. Medical Decision Making 1994; 14(2): 118–123. 10.1177/0272989X9401400204 8028464

[10] Meszaros JR, Asch DA, Baron J, Hershey JC, Kunreuther H, Schwartz-Buzaglo J. Cognitive processes and the decisions of some parents to forego pertussis vaccination for their children. Journal of Clinical Epidemiology 1996; 49(6): 697–703. 8656233 10.1016/0895-4356(96)00007-8

[11] Ritov I, Baron J. Protected values and omission bias. Organizational Behavior and Human Decision Processes 1999; 79(2): 79–94. 10.1006/obhd.1999.2839 10433898

[12] Johnson EJ, Hershey J, Meszaros J, Kunreuther H. Framing, probability distortions, and insurance decisions. Journal of Risk and Uncertainty 1993; 7(1): 35–51.

[13] Carroll GD, Choi JJ, Laibson D, Madrian BC, Metrick A. Optimal defaults and active decisions. The Quarterly Journal of Economics 2009; 124(4): 1639–1674. 20041043 10.1162/qjec.2009.124.4.1639 PMC2798815

[14] Dhingra N, Gorn Z, Kener A, Dana J. The default pull: An experimental demonstration of subtle default effects on preferences. Judgment & Decision Making 2012; 7(1): 69–76.

[15] Hayashi AT. Occasionally libertarian: Experimental evidence of self-serving omission bias. Journal of Law, Economics, and Organization 2013; 29(3): 711–733.

[16] Sunstein CR. Moral heuristics. Behavioral and brain sciences 2005; 28(4): 531–541. 10.1017/S0140525X05000099 16209802

[17] Bénabou R, Tirole J. Incentives and prosocial behavior. The American Economic Review 2006; 96(5): 1652–1678.

[18] Ellingsen T, Johannesson M. Pride and prejudice: The human side of incentive theory. The American Economic Review 2008; 98(3): 990–1008.

[19] Andreoni J, Bernheim BD. Social image and the 50–50 norm: A theoretical and experimental analysis of audience effects. Econometrica 2009; 77(5):1607–1636.

[20] Bénabou R, Tirole J. Identity, morals, and taboos: Beliefs as assets. The Quarterly Journal of Economics 2011; 126(2): 805–855. 22073409 10.1093/qje/qjr002

[21] Levitt SD, List JA. What do laboratory experiments measuring social preferences reveal about the real world? The Journal of Economic Perspectives 2007; 21(2): 153–174.

[22] Krupka EL, Weber RA. Identifying social norms using coordination games: Why does dictator game sharing vary? Journal of the European Economic Association 2013; 11(3): 495–524.

[23] Gill D, Prowse V. A novel computerized real effort task based on sliders. IZA Discussion Paper No. 5801 2011. https://papers.ssrn.com/sol3/papers.cfm?abstract_id=1877614.

[24] Greiner B. Subject Pool Recruitment Procedures: Organizing Experiments with ORSEE, Journal of the Economic Science Association 2015; 1(1): 114–125.

[25] Fischbacher U. z-Tree: Zurich toolbox for ready-made economic experiments. Experimental Economics 2007; 10(2): 171–178.

[26] Anderson CJ. The psychology of doing nothing: Forms of decision avoidance result from reason and emotion. Psychological Bulletin 2003; 129(1): 139–167. 12555797 10.1037/0033-2909.129.1.139

[27] Dana J, Weber R, Kuang JX. Exploiting moral wiggle room: Experiments demonstrating an illusory preference for fairness. Economic Theory 2007; 33(1): 67–80.

[28] List JA. On the interpretation of giving in dictator games. Journal of Political Economy 2007; 115(3): 482–493.

[29] Bardsley N. Dictator game giving: Altruism or artifact. Experimental Economics 2008; 11(2): 122–133.

[30] Dreber A, Ellingsen T, Johannesson M, Rand DG. Do people care about social context? Framing effects in dictator games. Experimental Economics 2013; 16(3): 349–371.

[31] Abadie A, Gay S. The impact of presumed consent legislation on cadaveric organ donation: a cross-country study. Journal of Health Economics 2006; 25(4): 599–620. 10.1016/j.jhealeco.2006.01.003 16490267

[32] Johnson EJ, Goldstein D. Do Defaults Save Lives? Science 2003; 302: 1338–1339. 10.1126/science.1091721 14631022

[33] Pichert D, Katsikopoulos KV. Green defaults: Information presentation and pro-environmental behaviour. Journal of Environmental Psychology 2008; 28(1): 63–73.

[34] Grossman Z. Strategic ignorance and the robustness of social preferences. Management Science 2014; 60(11): 2659–2665.

[35] Dana J, Cain DM, Dawes RM. What you don’t know won’t hurt me: Costly (but quiet) exit in dictator games. Organizational Behavior and Human Decision Processes 2006; 100(2): 193–201.

[36] Andreoni J, Rao JM, Trachtman H. Avoiding the ask: A field experiment on altruism, empathy, and charitable giving. Journal of Political Economy; Forthcoming.

[37] DellaVigna S, List JA, Malmendier U. Testing for altruism and social pressure in charitable giving. The Quarterly Journal of Economics 2012; 127(1): 1–56. 22448394 10.1093/qje/qjr050

[38] Lazear EP, Malmendier U, Weber RA. Sorting in experiments with application to social preferences. American Economic Journal: Applied Economics 2012; 4(1): 136–163.

[39] Ritov I, Baron J. Status-quo and omission biases. Journal of Risk and Uncertainty 1992; 5(1): 49–61.

[40] Gärtner M. Omission effects in trolley problems with economic outcomes. In Gärtner, Prosocial Behavior and Redistributive Preferences (Essay III) 2015. Stockholm: Departments of Economics, Stockholm University. http://su.diva-portal.org/smash/record.jsf?pid=diva2%3A862213&dswid=2627.

[41] Coffman LC. Intermediation reduces punishment (and reward). American Economic Journal: Microeconomics 2011; 3(4): 77–106.

[42] DeScioli P, Christner J, Kurzban R. The omission strategy. Psychological Science 2011; 22(4): 442–446. 10.1177/0956797611400616 21372326

[43] Cox J, Servátka M, Vadovič R. Status quo effects in fairness games: Reciprocal responses to acts of commission vs. acts of omission. Experimental Economics 2016.

