# Supplementary figures and images for: Is there an omission effect in prosocial behavior? A laboratory experiment on passive vs. active generosity
Source: PLoS One. 2017 Mar 1;12(3):e0172496. doi: 10.1371/journal.pone.0172496 (PMC5383002; doi:10.1371/journal.pone.0172496)

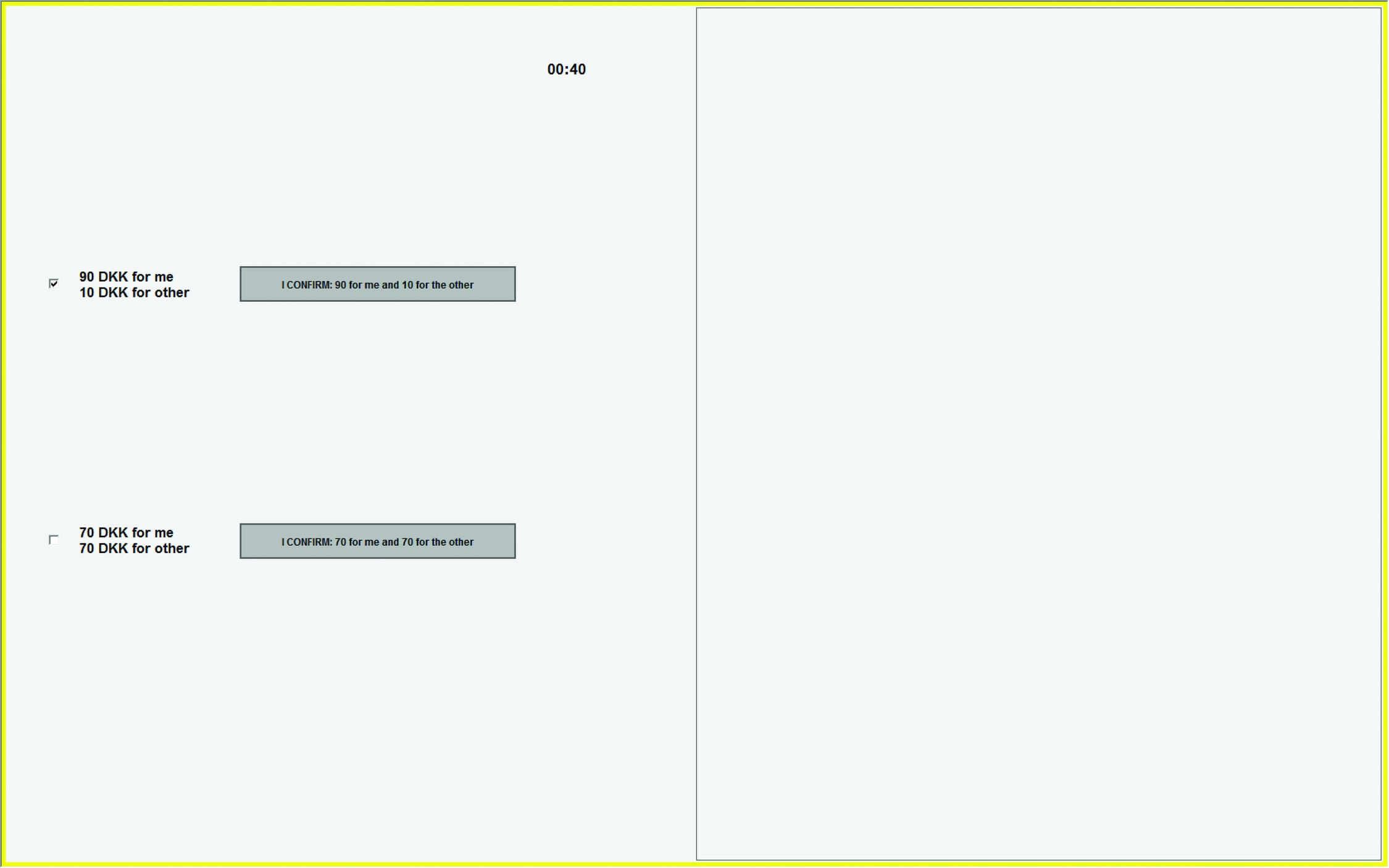

Supplement: S1 Fig — (TIF) [file pone.0172496.s001.tif]

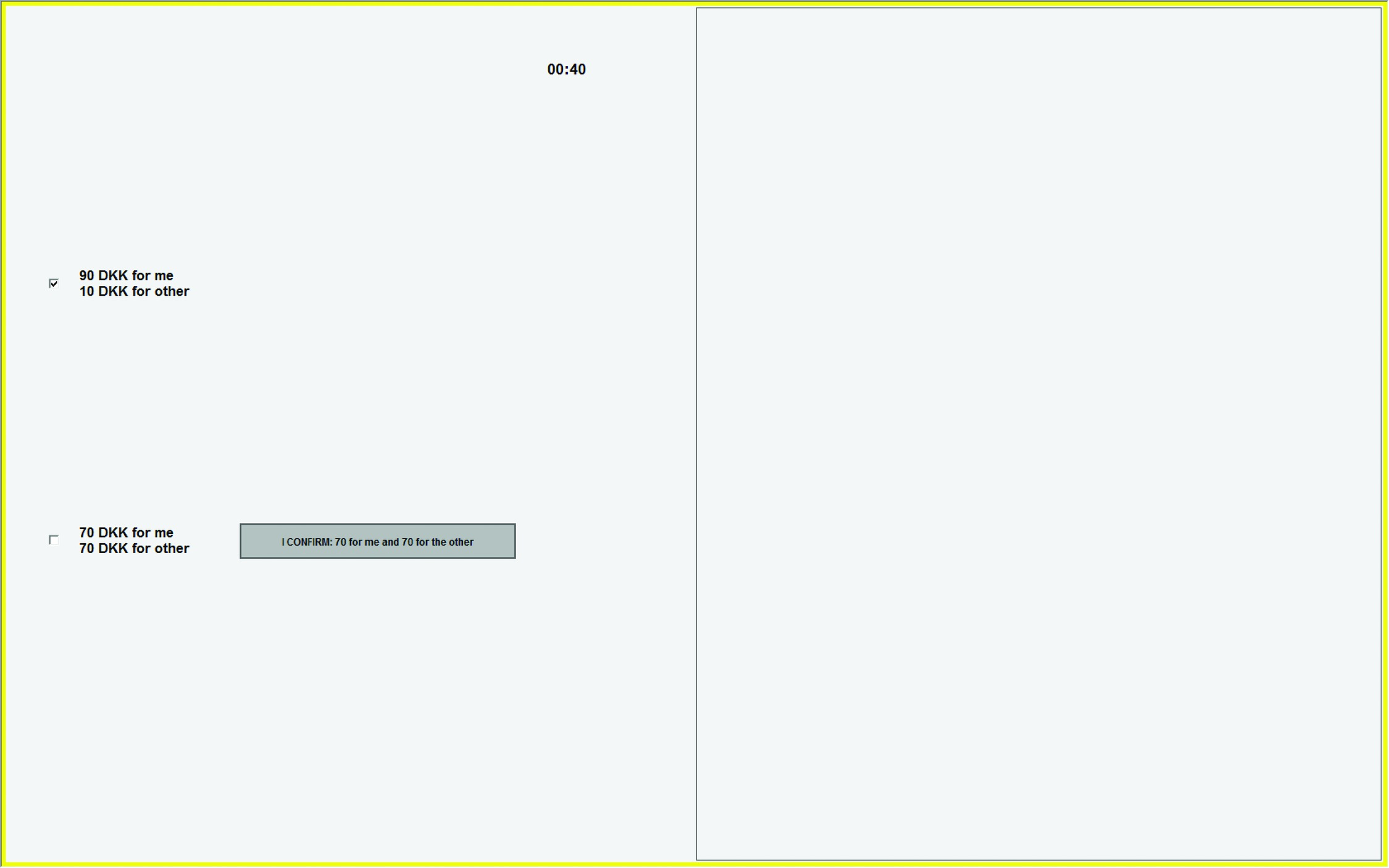

Supplement: S2 Fig — (TIF) [file pone.0172496.s002.tif]

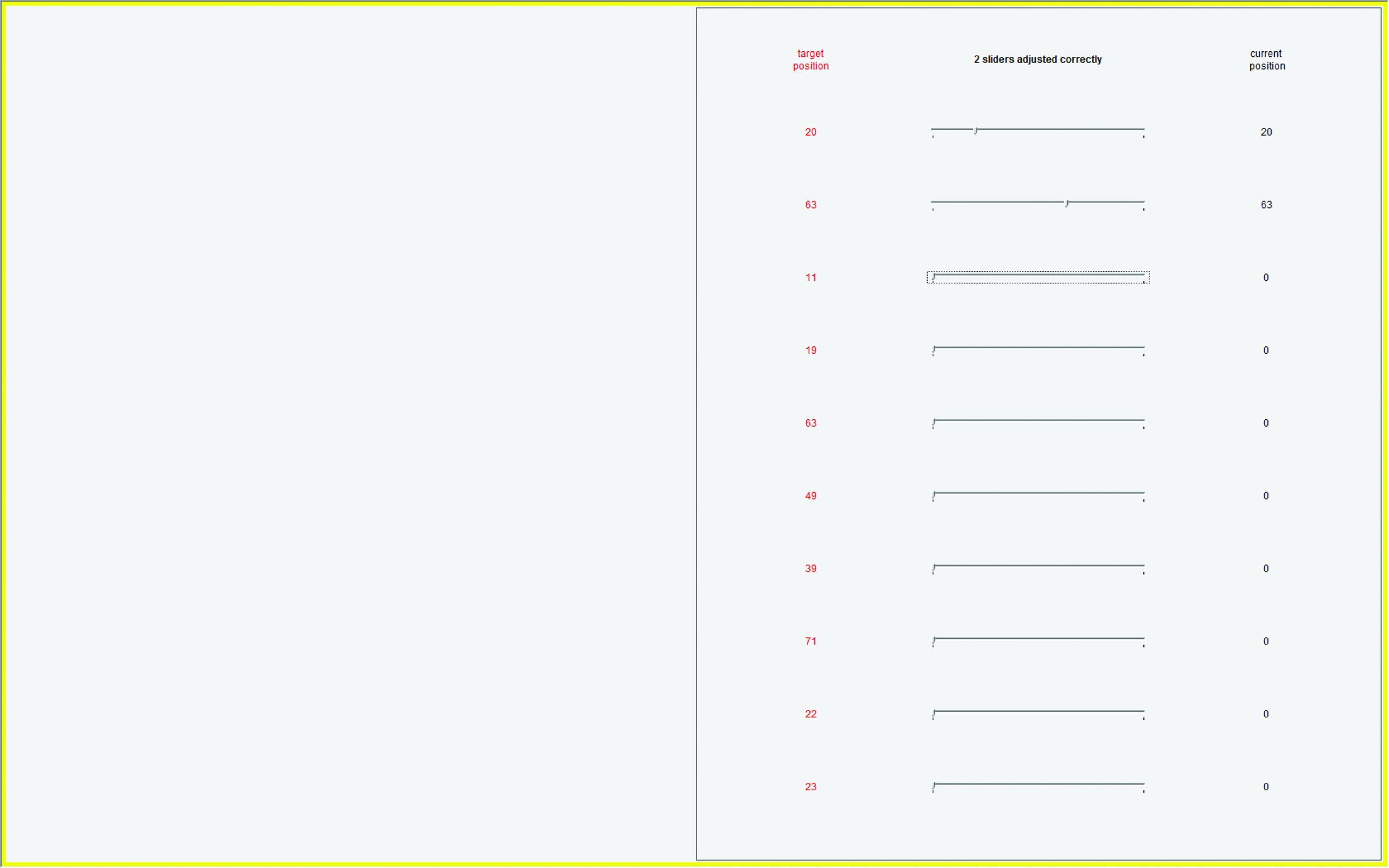

Supplement: S3 Fig — (TIF) [file pone.0172496.s003.tif]
