# Supplementary material for: Is there an omission effect in prosocial behavior? A laboratory experiment on passive vs. active generosity
Source: PLoS One. 2017 Mar 1;12(3):e0172496. doi: 10.1371/journal.pone.0172496 (PMC5383002; doi:10.1371/journal.pone.0172496)
Supplement: S1 File — (PDF) [file pone.0172496.s004.pdf]

SUPPORTING INFORMATION S1

Additional tables

Table A All allocation choices.

Choice nr.	π_i	π_j	$\hat{\pi}_i$	$\hat{\pi}_j$	$\frac{\hat{\pi}_j - \pi_j}{(\pi_i - \hat{\pi}_i)}$	$\pi_i - \pi_j$	$\hat{\pi}_i - \hat{\pi}_j$
1	90	10	70	70	3	80	0
2	75	75	90	60	1	0	30
3	140	10	75	75	1	130	0
4	125	25	150	0	1	100	150
5	150	0	90	60	1	150	30
6	125	40	135	0	4	85	135
7	110	40	70	80	1	70	-10
8	70	80	140	10	1	-10	130
9	100	25	75	100	3	75	-25
10	90	90	110	10	4	0	100
11	40	80	60	60	1	-40	0
12	50	50	30	110	3	0	-80
13	50	100	50	50	-	-50	0
14	25	125	0	0	-5	-100	0

Note: π_i denotes payoff to the dictator and π_j denotes payoff to the recipient. Each allocation involves a tradeoff between the two payoff vectors (π_i, π_j) and $(\hat{\pi}_i, \hat{\pi}_j)$. The ratio $(\hat{\pi}_j - \pi_j)/(\pi_i - \hat{\pi}_i)$ indicates the "relative price" of giving, i.e. the amount of DKK the recipient gains (loses) for every DKK the dictator loses (gains). $\pi_i - \pi_j$ and $\hat{\pi}_i - \hat{\pi}_j$ indicates the inequality of payoffs between the dictator and the recipient in the two payoff vectors (a positive number indicates that the dictator is ahead and a negative number indicates that the dictator is behind). All subjects face choice 1 as their first choice. The order of choices 2-14 is randomized.

Table B Participant characteristics.

	All	Commission treatment	Omission treatment	Difference	*p*-value
Age	25.595	25.320	25.870	-0.550	0.196
	(4.253)	(4.025)	(4.463)		
Female	0.480	0.465	0.495	-0.030	0.550
	(0.500)	(0.500)	(0.501)		
Danish	0.258	0.240	0.276	-0.036	0.419
	(0.438)	(0.428)	(0.448)		
Full time student	0.778	0.758	0.798	-0.040	0.334
	(0.416)	(0.430)	(0.403)		
Economics course(s)	0.610	0.635	0.585	0.050	0.305
taken	(0.488)	(0.483)	(0.494)		

Note: Standard deviations in parentheses. *N*=400. All variables except for *Age* are binary. The *p*-value for the age difference across treatments is obtained using a two-sided, two-sample t-test. All other p-values are obtained using chi-squared tests.

Table C Treatment effects in all 14 allocation choices

Choice no.	π_i	π_j	$\hat{\pi}_i$	$\hat{\pi}_j$	Share of default choices: Default (π_i, π_j): Commission	Omission	Omission – Commission: Effect size	p	Default $(\hat{\pi}_i, \hat{\pi}_j)$: Commission	Omission	Omission – Commission: Effect size	p
1	90	10	70	70	0.438	0.467	0.028	(0.691)	0.638	0.526	-0.112	(0.109)
2	75	75	90	60	0.390	0.330	-0.060	(0.380)	0.653	0.670	0.017	(0.797)
3	140	10	75	75	0.684	0.648	-0.035	(0.607)	0.449	0.339	-0.110	(0.107)
4	125	25	150	0	0.535	0.467	-0.068	(0.334)	0.543	0.583	0.040	(0.582)
5	150	0	90	60	0.538	0.656	0.119	(0.096)	0.442	0.404	-0.038	(0.575)
6	125	40	135	0	0.651	0.558	-0.093	(0.198)	0.333	0.448	0.115	(0.091)
7	110	40	70	80	0.769	0.768	-0.002	(0.979)	0.222	0.257	0.035	(0.570)
8	70	80	140	10	0.378	0.258	-0.120	(0.082)	0.733	0.692	-0.042	(0.502)
9	100	25	75	100	0.618	0.690	0.073	(0.263)	0.366	0.391	0.025	(0.727)
10	90	90	110	10	0.623	0.514	-0.109	(0.110)	0.432	0.452	0.020	(0.789)
11	40	80	60	60	0.043	0.061	0.019	(0.561)	0.990	0.941	-0.049	(0.054)
12	50	50	30	110	0.917	0.806	-0.110	(0.022)	0.116	0.168	0.052	(0.308)
13	50	100	50	50	0.592	0.632	0.040	(0.556)	0.500	0.532	0.032	(0.658)
14	25	125	0	0	0.930	0.894	-0.036	(0.370)	0.075	0.123	0.047	(0.267)

Note: π_i denotes payoff to the dictator and π_j denotes payoff to the recipient. Each allocation involves a tradeoff between the two payoff vectors (π_i, π_j) and $(\hat{\pi}_i, \hat{\pi}_j)$. All subjects face choice 1 as their first choice. The order of choices 2-14 is randomized within subject.
