# Supplementary material for: Is there an omission effect in prosocial behavior? A laboratory experiment on passive vs. active generosity
Source: PLoS One. 2017 Mar 1;12(3):e0172496. doi: 10.1371/journal.pone.0172496 (PMC5383002; doi:10.1371/journal.pone.0172496)
Supplement: S2 File — (PDF) [file pone.0172496.s005.pdf]

SUPPORTING INFORMATION S2

Ethics statement

The following ethics statement applies to the experimental studies in "Is there an omission effect in prosocial behavior? A laboratory experiment on passive vs. active generosity" that uses human subjects in experiments studying how individuals allocate money.

Both authors of the study are and were, at the time that the study was conducted, employed as researchers at Swedish universities. According to guidelines from the Swedish research council, approval from an ethics committee is not required for behavioral studies that do not fall under the Swedish law concerning the Ethical Review of Research Involving Humans (SFS 2003:460). Please find a link to an English version of this law attached. The purpose of the law is to protect individuals and human dignity when research is conducted. In particular, Sections 3 and 4 of the law specify that ethical approval is required if the study collects sensitive personal data (such as involving and handling of social security numbers or criminal records), conducts interventions that may be physically or mentally harmful, or collects biological material. The following is an excerpt from the law that states these conditions:

Section 3

This law shall apply to research that includes the handling of:

1. Sensitive personal data pursuant to Section 13 of the Personal Data Act (1998:204), or

2. Personal data regarding violations of law that include crimes, judgments in criminal cases, penal law sanctions, or administrative deprivation of liberty, as defined in Section 21 of the Personal Data Act.

Section 4

In addition to that which follows from Section 3, the law shall apply to research that:

1. Subjects a research subject to a physical intervention,

2. Is performed according to a method with the purpose of affecting a research person physically or mentally, or includes an apparent risk of injuring the research subject either physically or mentally

3. Relates to studies of biological material that has been taken from a living person, and can be traced to that person

4. Constitutes a physical intervention on a deceased person, or

5. Relates to studies of biological material that has been taken from a deceased person for medical purposes, and can be traced to that person.

The experiments in our study did not include the handling of information according to items 1-2 of Section 3, or any interventions according to items 1-5 of Section 4. All studies (both in the laboratory and online) clearly indicated to the subjects that the study was conducted for the purposes of research and that the data would be analyzed anonymously. The collected data cannot in any way be connected to personal identifiers, such as social security numbers, and do not contain other sensitive personal information. All subjects participated voluntarily in the studies and were at least 18 years old. Participants in the laboratory experiment had voluntarily signed up to participate in the subject pool at the Centre for Experimental Economics (http://www.econ.ku.dk/cee/participate/), and thus been informed of the rules that the laboratory applies. Similarly, participants in the online experiment had voluntarily registered as workers at the online labor market and then actively chosen the to participate in the current study. All studies were conducted under full-disclosure, meaning that there was no deceit. All treatments were non-invasive and subject payments were made as indicated to subjects in the study.

For these reasons, we did not ask the Central Ethical Review Board (Etikprövningsnämnden) for a written waiver or collected explicit written informed consent from the subjects. There has been no such praxis present in our field of study in Sweden. The decision in Sweden is and has been the discretion of the individual researcher and research departments. The Department of Economics at Stockholm University and the Department of Economics at the Stockholm School of Economics (Sandberg's employer at the time of the study) did not consider the study being eligible for review or that it required collecting informed consent in line with the Swedish legislation on ethical review.

The Ethical Review Act: http://www.epn.se/media/2348/the_ethical_review_act.pdf
