# Supplementary material for: Is there an omission effect in prosocial behavior? A laboratory experiment on passive vs. active generosity
Source: PLoS One. 2017 Mar 1;12(3):e0172496. doi: 10.1371/journal.pone.0172496 (PMC5383002; doi:10.1371/journal.pone.0172496)
Supplement: S3 File — (PDF) [file pone.0172496.s006.pdf]

SUPPORTING INFORMATION S3

Results for fair default and Hypothesis 2 (Experiment 1)

First allocation choice

Our second hypothesis addresses the concern that an observed omission effect under selfish defaults may reflect a more general omission effect. This would imply a similar omission effect also under fair defaults. When the fair allocation (70,70) is presented as the default option, the share of selfish choices is 36.2% in the commission treatment and 47.4% in the omission treatment. Thus, rather than showing an omission effect, subjects display a tendency, albeit not statistically significant ($\chi^2(1)$ = 2.57, p = 0.109), to be *less* prone to implement the fair default allocation by omission rather than by commission. If anything, this could indicate that prosocial choices contribute less to a subject's positive self- or social image if they are implemented by omission rather than by commission. Given this result, and given that we find no significant omission effect under selfish defaults, we can rule out the existence of a general omission effect. Nevertheless, it may be interesting to investigate whether omissions have a significantly different effect on fair choices as compared to selfish choices. The first regression in Table A provides such a test. The coefficient of the interaction term indicates that the absolute difference in omission effects between the (90,10) default condition and the (70,70) default condition is 14 percentage points, but not statistically significant. Hence, we cannot reject the null hypothesis of no difference between the two default conditions.

All allocation choices

The omission effect under non-selfish defaults, pooling all allocation choices, is given by the coefficient of the indicator variable *Omission treatment* in Models 2-5 in Table A. This effect tends to be negative and is statistically not significant at the 5 percent level in all models. In Model 2, when we exclude choices that involve behindness, the effect is marginally significant at the 10 percent level. Thus, in line with the results from the first allocation choice, we find no tendency for a general omission effect when looking at all choices pooled. Further, the difference in omission effects between choices with a selfish and a non-selfish default, as indicated by the interaction term, is not significant at the 5 percent level in all models. This difference is significant at the 10 percent level only in Model 3, showing a difference of 12.9 percentage points.[1]

[1] When including the observations that have been dropped due to a failure to make an active choice in the commission treatment, some significance levels in Model 3 of Table A change slightly. Under the assumption

Table A Treatment and default effects on the propensity to choose default, all choices pooled

	Model 1	Model 2	Model 3	Model 4	Model 5
Omission treatment	-0.112<br>(0.070)	-0.050<br>(0.033)	-0.083*<br>(0.044)	0.005<br>(0.032)	-0.058<br>(0.041)
Selfish default	-0.200***<br>(0.071)	0.279***<br>(0.038)	-0.016<br>(0.052)	0.467***<br>(0.031)	0.066<br>(0.045)
Omission treatment X Selfish default	0.140<br>(0.100)	0.054<br>(0.054)	0.129*<br>(0.072)	-0.024<br>(0.046)	0.078<br>(0.065)
Constant	0.638***<br>(0.048)	0.387***<br>(0.023)	0.531***<br>(0.031)	0.290***<br>(0.022)	0.488***<br>(0.029)
R^2	0.02	0.10	0.01	0.21	0.01
N (choices)	394	5,132	2,765	2,370	1,971
Choices included	First choice	Selfish vs. non-selfish	Selfish vs. non-selfish (not behind)	Selfish vs. behind	Selfish vs. efficient

Note: OLS regressions. The sample is restricted to choices where the default option is non-selfish (not strictly payoff dominant for the dictator). Dependent variable: = 1 if default chosen, = 0 otherwise. *Model 1* only includes the first choice between (90,10) and (70,70). *Model 2* includes all allocation choices except choice number 13 which has no strictly payoff dominant option for the dictator. *Model 3* includes choices 1, 2, 3, 4, 5, 6 and 10. *Model 4* includes choices 7, 8, 9, 11, 12 and 13. *Model 5* includes choices 1, 6, 9, 10 and 12. See Table A in S1 File for a list of all choices. Standard errors are clustered on participant in all models except for *Model 1*. * p<0.1; ** p<0.05; *** p<0.01

that these choices would have been non-selfish, the interaction term and the coefficient of *Omission treatment* become significant at the 5 percent level. Under the assumption that these choices would have been default choices, the coefficient of *Omission treatment* becomes significant at the 5 percent level.
