# Supplementary material for: Is there an omission effect in prosocial behavior? A laboratory experiment on passive vs. active generosity
Source: PLoS One. 2017 Mar 1;12(3):e0172496. doi: 10.1371/journal.pone.0172496 (PMC5383002; doi:10.1371/journal.pone.0172496)
Supplement: S4 File — (PDF) [file pone.0172496.s007.pdf]

SUPPORTING INFORMATION S4

Structural estimation

In this section, we exploit the panel data nature of our data set to test our first hypothesis by classifying subjects into different social preference types. In particular, we investigate if the distribution of types differs between the omission and the commission treatment, given a selfish default. Thus, we restrict the sample to all choices with a default that is payoff-dominant for the dictator. The structural estimation provides a useful addition to our other estimates as it takes both inequity aversion and social welfare preferences into account in the same model. While our previous analyses are restricted to comparing average selfishness rates across treatments, the structural estimation allows for a more multifaceted distribution of individual social preferences.

We assume that individual *i*'s preferences take the following theoretical form, similar to the specification in [1] and [2]:

$$U_i(\pi_i,\, \pi_j) = \beta_i \pi_i + \rho_i r(\pi_j - \pi_i) + \sigma_i s(\pi_j - \pi_i),$$

where

$$r = 1\ if\ \pi_i > \pi_j, r = 0\ otherwise,$$

$$s = 1\ if\ \pi_i < \pi_j, s = 0\ otherwise,$$

denoting individual *i*'s payoff by π_i and the other individual's payoff by π_j. The parameter β_i captures the weight individual *i* places on her own income, and the parameter ρ_i [σ_i] captures the weight that *i* places on relative income (π_j - π_i) while *i* earns more [less] than *j*. Thus, given β_i>0, for a completely *selfish* individual, who only cares about maximizing her own payoff, we would observe $\rho_i = \sigma_i$=0. For an *inequality averse* individual, who prefers to minimize differences in income between herself and others, we would observe ρ_i>0 and σ_i<0. This utility function is simple and parsimonious while still allowing for identifying a range of potentially important social motivations, such as inequity aversion and social welfare preferences, through a few binary allocation choices. Note that our set of binary choices varies these motives in a way that allows us to calibrate the function. This utility function is also commonly used in previous literature, and thus well suited for comparison purposes.

The number of decisions per subject in our data set is too small to estimate β_i, ρ_i and σ_i individually for each subject. Instead, we estimate a latent class conditional logit model, allowing for a finite number *C* of classes (or "types") in the population. Each subject is assumed to belong to a class *c*, and we allow preference parameters β_c, ρ_c, and σ_c to vary across, but not within, classes. If an individual *i* belongs to class *c* and faces *T* different choices with two alternatives in each choice, the probability of observing a particular sequence of choices is

$$P_i\left(\beta_{c,}\,\rho_{c,}\,\sigma_c\right) = \prod_{t=1}^{T}\prod_{k=1}^{2}\left(\frac{\exp\left(U_{ikt}\left(\pi_{ikt},\,\pi_{jkt};\,\beta_{c,}\,\rho_{c,}\,\sigma_c\right)\right)}{\sum_{m=1}^{2}\exp\left(U_{i\,mt}\left(\pi_{imt},\,\pi_{jmt};\,\beta_{m,}\,\rho_{m,}\,\sigma_m\right)\right)}\right)^{y_{ikt}},$$

where y_{ikt} is our dependent variable that takes the value of 1 if agent *i* chooses choice alternative *k* in choice *t*, and 0 otherwise.

H_c denotes the probability of belonging to a class *c*, and is specified as

$$H_c(\boldsymbol{\theta}) = \frac{\exp(\boldsymbol{\theta_c z_i})}{1+\sum_{l}^{C-1}\exp(\boldsymbol{\theta_l z_i})},$$

where $\boldsymbol{\theta} = (\boldsymbol{\theta_1}, \boldsymbol{\theta_2}, \ldots, \boldsymbol{\theta_{C-1}})$ are class membership parameters that are estimated along with the preference parameters and $\boldsymbol{\theta_C}$ is normalized to zero. The individual-specific characteristics $\boldsymbol{z_i}$ include an indicator for the treatment group (omission or commission) and a constant. Thus, class membership varies with treatment status.

The log-likelihood for this model is

$$\ln L\,(\boldsymbol{\beta}, \boldsymbol{\rho}, \boldsymbol{\sigma}, \boldsymbol{\theta}) = \sum_{i=1}^{N} ln \sum_{c=1}^{C} H_c(\boldsymbol{\theta}) P_i\left(\beta_{c,}\,\rho_{c,}\,\sigma_c\right),$$

where $\boldsymbol{\beta} = (\beta_1, \beta_2, \ldots, \beta_C)$, $\boldsymbol{\rho} = (\rho_1, \rho_2, \ldots, \rho_C)$ and $\boldsymbol{\sigma} = (\sigma_1, \sigma_2, \ldots, \sigma_C)$. The model is estimated using the *lclogit* package and the *glamm* package for Stata [3,4].

To determine the optimal number of classes *C*, we estimate the model using 2-10 numbers of classes and compute the Bayesian information criterion (BIC) and the Akaike information criterion (AIC) for each specification. Both information criteria suggest a

specification with three classes. Using three classes, the model estimates the nine preference parameters, $(\beta_{1,}\ \rho_{1,}\ \sigma_1)$, $(\beta_{2,}\ \rho_{2,}\ \sigma_2)$, $(\beta_{3,}\ \rho_{3,}\ \sigma_3)$.

In Table A, we report the estimated preference parameters for each of the three types, and the share of subjects that is estimated to be of each type, both overall and for each treatment. Following the above description of the preference parameters, we can roughly characterize the first type as inequality averse and the second type as selfish. The third type is best described as having maxi-min social preferences, only caring about the income of the other person when ahead and only about her own income when behind. Overall, the share of inequality averse types is 43.8%, the share of selfish types is 39.5% and the share of maxi-min types is 16.7%. A joint test of whether the shares of types differ between the omission and the commission treatment shows that there is no significant treatment effect ($\chi^2(2)$ = 1.40, p = 0.4975). Hence, given a selfish default option, we find no evidence that the distinction between active and passive choices has an independent effect on an individual's prosocial behavior. Consequently, even when taking into account that there is a distribution of individual social preferences, and that individual choices might be classified in other ways than merely selfish vs. non-selfish, we can confirm the findings from our main analyses.

Table A Latent class model estimates for defaults with $\pi_i > \hat{\pi}_i$

	Type 1	Type 2	Type 3
β	0.086***	0.192***	0.060***
	(0.006)	(0.038)	(0.014)
ρ	0.039***	0.018*	0.061***
	(0.003)	(0.009)	(0.010)
σ	-0.007**	-0.013	0.005
	(0.003)	(0.013)	(0.004)
N	2576 choices by 397 participants		
Proportion of types:			
Overall	43.8%	39.5%	16.7%
Commission treatment	47.0%	36.3%	16.7%
Omission treatment	40.7%	42.5%	16.8%

Dependent variable: Binary variable indicating whether allocation was chosen or not. Standard errors in parentheses. Significance levels are denoted by * $p < 0.05$, ** $p < 0.01$, *** $p < 0.001$. Parameters are estimated using the expectation maximization algorithm. The model includes all allocation trade-offs except choice 13 which has no strictly payoff dominant option for the dictator.

References

1. Charness G, Rabin M. Understanding social preferences with simple tests. The Quarterly Journal of Economics 2006; 117(3): 817-869.

2. Fehr E, Schmidt KM. A theory of fairness, competition, and cooperation. The Quarterly Journal of Economics 1999; 114(3): 817-868.

3. Pacifico D, Yoo H. lclogit: A Stata command for fitting latent-class conditional logit models via the expectation-maximization algorithm. The Stata Journal 2013; 13(3): 625-639.

4. Skrondal A, Rabe-Hesketh S. Generalized latent variable modeling: Multilevel, longitudinal, and structural equation models. Boca Raton, FL: Chapman & Hall/CRC; 2004.
