# Supplementary material for: Is there an omission effect in prosocial behavior? A laboratory experiment on passive vs. active generosity
Source: PLoS One. 2017 Mar 1;12(3):e0172496. doi: 10.1371/journal.pone.0172496 (PMC5383002; doi:10.1371/journal.pone.0172496)
Supplement: S5 File — (PDF) [file pone.0172496.s008.pdf]

SUPPORTING INFORMATION S5

Robustness checks

Participants in the commission treatment failed to make an active choice in 2.6% of all choice occasions. In our main analyses, we exclude these observations. In this section, we test the robustness of our main results by reproducing Table 3 including these observations. In Table A, we assume that these observations would have been selfish choices (i.e. default choices), and in Table B we assume that they would have been non-selfish choices (i.e. not default choices).

Table A Treatment effect on the propensity to choose the selfish default, interpreting failure to choose in the commission treatment as a selfish choice.

	Model 1	Model 2	Model 3	Model 4	Model 5
Omission treatment	0.010	-0.004	0.037	-0.025	0.010
	(0.072)	(0.030)	(0.041)	(0.030)	(0.039)
Constant	0.457***	0.674***	0.523***	0.763***	0.564***
	(0.052)	(0.020)	(0.029)	(0.019)	(0.028)
R^2	0.00	0.00	0.00	0.00	0.00
N (choices)	197	2,605	1,359	1,242	1,009
Choices included:	First choice	Selfish vs. non-selfish	Selfish vs. non-selfish (not behind)	Selfish vs. behind	Selfish vs. efficient

Note: OLS regressions. The sample is restricted to choices where the default option is selfish (strictly payoff dominant for the dictator). Dependent variable: = 1 if default chosen, = 0 otherwise. *Model 1* only includes the first choice between (90,10) and (70,70). *Model 2* includes all allocation choices except choice number 13 which has no strictly payoff dominant option for the dictator. *Model 3* includes choices 1, 2, 3, 4, 5, 6 and 10. *Model 4* includes choices 7, 8, 9, 11, 12 and 13. *Model 5* includes choices 1, 6, 9, 10 and 12. See Table A in S1 File for a list of all choices. Standard errors are clustered on participant in all models except for *Model 1*. * p<0.1; ** p<0.05; *** p<0.01.

Table B Treatment effect on the propensity to choose the selfish default, interpreting failure to choose in the commission treatment as a non-selfish choice.

	Model 1	Model 2	Model 3	Model 4	Model 5
Omission treatment	0.043	0.019	0.055	-0.001	0.032
	(0.071)	(0.031)	(0.041)	(0.031)	(0.039)
Constant	0.424***	0.651***	0.505***	0.740***	0.542***
	(0.052)	(0.021)	(0.029)	(0.021)	(0.028)
R^2	0.00	0.00	0.00	0.00	0.00
N (choices)	197	2,605	1,359	1,242	1,009
Choices included:	First choice	Selfish vs. non-selfish	Selfish vs. non-selfish (not behind)	Selfish vs. behind	Selfish vs. efficient

Note: OLS regressions. The sample is restricted to choices where the default option is selfish (strictly payoff dominant for the dictator). Dependent variable: = 1 if default chosen, = 0 otherwise. *Model 1* only includes the first choice between (90,10) and (70,70). *Model 2* includes all allocation choices except choice number 13 which has no strictly payoff dominant option for the dictator. *Model 3* includes choices 1, 2, 3, 4, 5, 6 and 10. *Model 4* includes choices 7, 8, 9, 11, 12 and 13. *Model 5* includes choices 1, 6, 9, 10 and 12. See Table A in S1 File for a list of all choices. Standard errors are clustered on participant in all models except for *Model 1*. * p<0.1; ** p<0.05; *** p<0.01.
