# Supplementary material for: Is there an omission effect in prosocial behavior? A laboratory experiment on passive vs. active generosity
Source: PLoS One. 2017 Mar 1;12(3):e0172496. doi: 10.1371/journal.pone.0172496 (PMC5383002; doi:10.1371/journal.pone.0172496)

**SUPPORTING INFORMATION S6**

**Experimental instructions for Experiment 1**

## General instructions

Welcome to this experiment! Please read these instructions, and the instructions on your screen, thoroughly. Do not talk to other participants during the experiment. Whenever you have any questions, please raise your hand and wait for one of us to come to you.

The experiment consists of two parts: *Part 1* and *Part 2*. These instructions describe Part 1. Information about Part 2 will follow once you have completed Part 1.

You can earn money in this experiment. All amounts stated in the experiment are in Danish kroner (DKK) and your earnings will be paid privately in cash at the end of the experiment. You are only paid for tasks that you completed according to the instructions given to you. **Your decisions and the decisions of other participants will remain anonymous.**

**Part 1 of this experiment consists of two different types of tasks: the *slider task* and the *distribution task*. It will take about 20 minutes. The size of your reward from this part will depend solely on your decisions.**

On the following pages, we describe both tasks, and give an overview of the structure of the experiment. After reading the instructions, you will have time to practice both tasks on the screen. We will also ask a number of control questions on the screen to make sure you understand the instructions.

## Instructions for slider task

The slider task provides a set of sliders on the right side of your screen.

You can adjust each slider to any position between 0 and 100 by pressing the slider with your mouse and dragging it to the desired position. There is one number at each end of a slider. The black number to the right tells you the current position of the slider. The red number to the left tells you a target position.

**A slider is correctly adjusted, when the current position is equal to the target position.** For example, the upper slider in the picture below has the current position of zero and a target position of 50. The lower slider in the picture shows the same slider when it is adjusted correctly – that is, when the current position is equal to the target position.

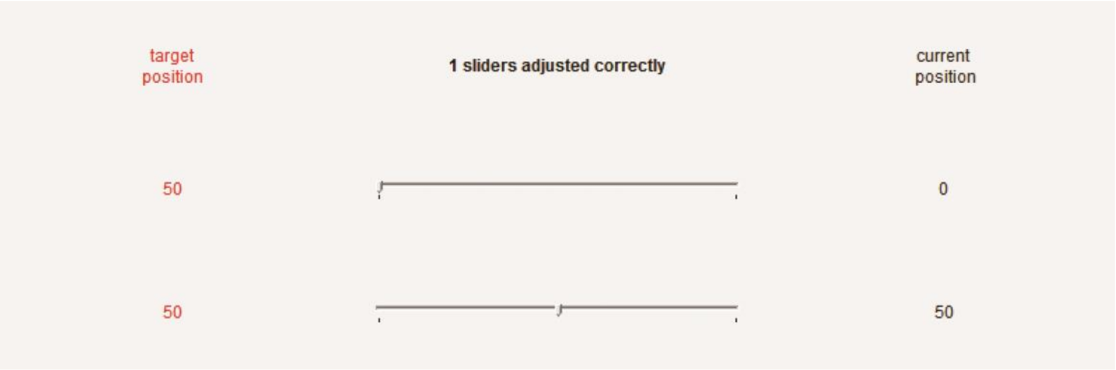


The sliders will be on your screen throughout part 1 of the experiment. As soon as one set of sliders is correctly adjusted, a new set of sliders will appear. Thus, you can adjust the sliders at any time and you can solve as many sliders as you like for the duration of the experiment. A counter on top of the sliders tells you how many sliders you adjusted correctly.

**You earn 0.01 DKK (1 øre) for each slider that is correctly adjusted.** At the end of the experiment, you will be paid for all the sliders you have correctly adjusted throughout the experiment.

## Instructions for distribution task

*a. For participants in the commission treatment*

The distribution task consists of several decisions. **Each decision will appear for 40 seconds on the left side of the screen, as indicated by a timer**. When the time on the timer has run out, the decision will disappear.

For each decision, you will be randomly paired with another participant, who is in this room and who participates in the slider task. You can choose one of two different distributions of money between you and the other participant.

When a decision appears on the screen, you will see two alternatives with checkboxes next to them. Each alternative states an amount of crowns that will be given to you and an amount that will be given to the other participant. One of the two checkboxes has already been checked at random.

For example, a decision between an alternative that gives 100 DKK to you and 100 DKK to the other, and an alternative that gives 200 DKK to you and 200 DKK to the other, where the first alternative has already been checked, would look like this:

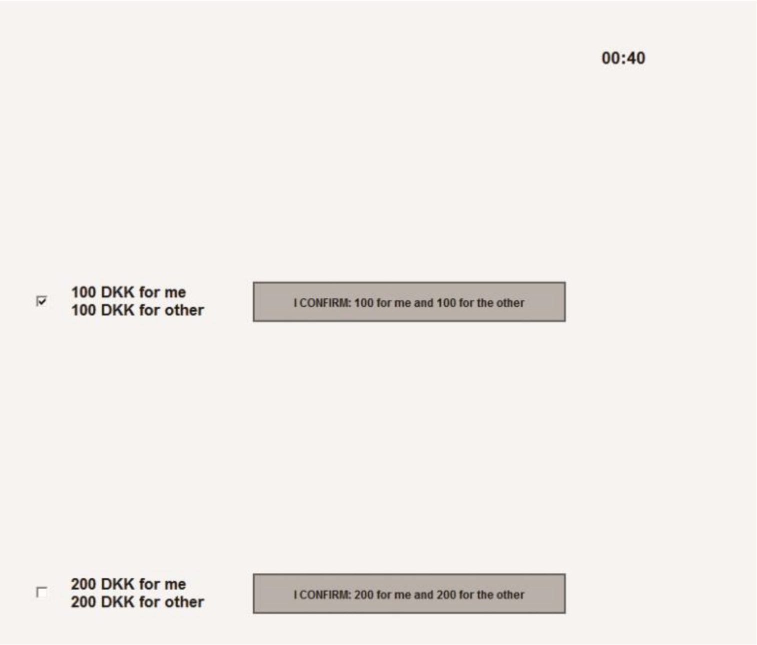


**To choose one of the two alternatives, the checkbox next to the alternative needs to be checked and the button stating the alternative needs to be pressed for confirmation**. If this is done correctly, the button will turn red, and you can no longer change your decision.

Note that the distribution task will not disappear before the time has run down, even if you have pressed a button.

For each new decision you face in the distribution task, you will be paired with a new participant. **At the end of the experiment, one of the decisions will be selected at random, and you and the other participant will be paid the amounts stated in the alternative that you chose.**

*b. For participants in the omission treatment*

The distribution task consists of several decisions. **Each decision will appear for 40 seconds on the left side of the screen, as indicated by a timer**. When the time on the timer has run out, the decision will disappear.

For each decision, you will be randomly paired with another participant, who is in this room and who participates in the slider task. You can choose one of two different distributions of money between you and the other participant.

When a decision appears on the screen, you will see two alternatives with checkboxes next to them. Each alternative states an amount of crowns that will be given to you and an amount that will be given to the other participant. One of the two checkboxes has already been checked at random.

For example, a decision between an alternative that gives 100 DKK to you and 100 DKK to the other, and an alternative that gives 200 DKK to you and 200 DKK to the other, where the first alternative has already been checked, would look like this:

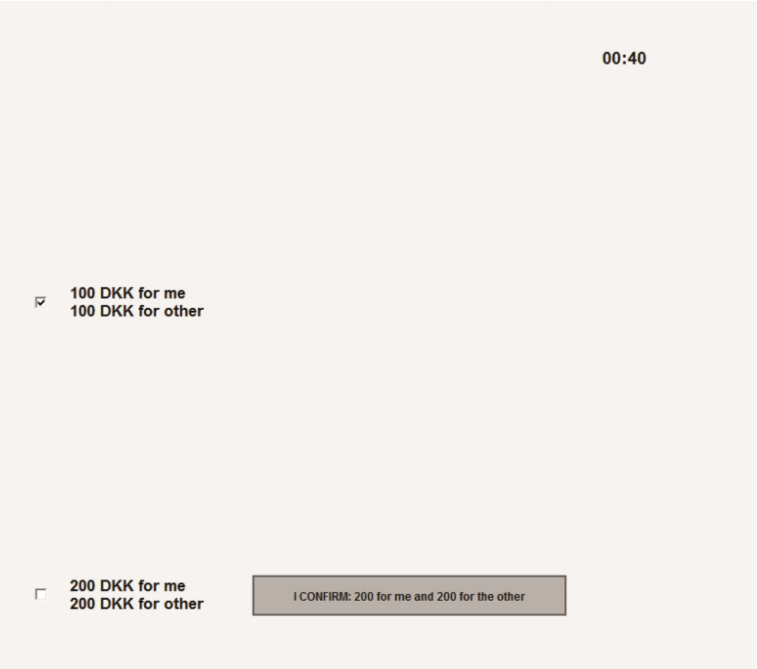


**The alternative that is already checked will be selected automatically when time has run out. To choose the other alternative, the checkbox next to that alternative needs to be checked and the button stating that alternative needs to be pressed for confirmation.** If this is done correctly, the button will turn red, and you can no longer change your decision.

Note that the distribution task will not disappear before the time has run down, even if you have pressed a button.

For each new decision you face in the distribution task, you will be paired with a new participant. **At the end of the experiment, one of the decisions will be selected at random, and you and the other participant will be paid the amounts stated in the alternative that you chose.**

## Overview of the experiment (example from commission treatment)

Below is a sketch of how Part 1 of the experiment evolves over time.

Throughout the experiment, there will always be sliders you can solve on the right side of the screen. From time to time, distribution task decisions will appear on the left side of the screen. When time has run down on the timer of a distribution task decision, that decision will disappear. In between the different decisions, the left side of the screen will be blank.

Please note: The slider task will always be present and the sliders can be adjusted at any time. Your adjustments to the sliders remain even when the distribution task appears or disappears.

How many sliders you or other participants solve does *not* have an influence on the amounts that you will face in the distribution task.

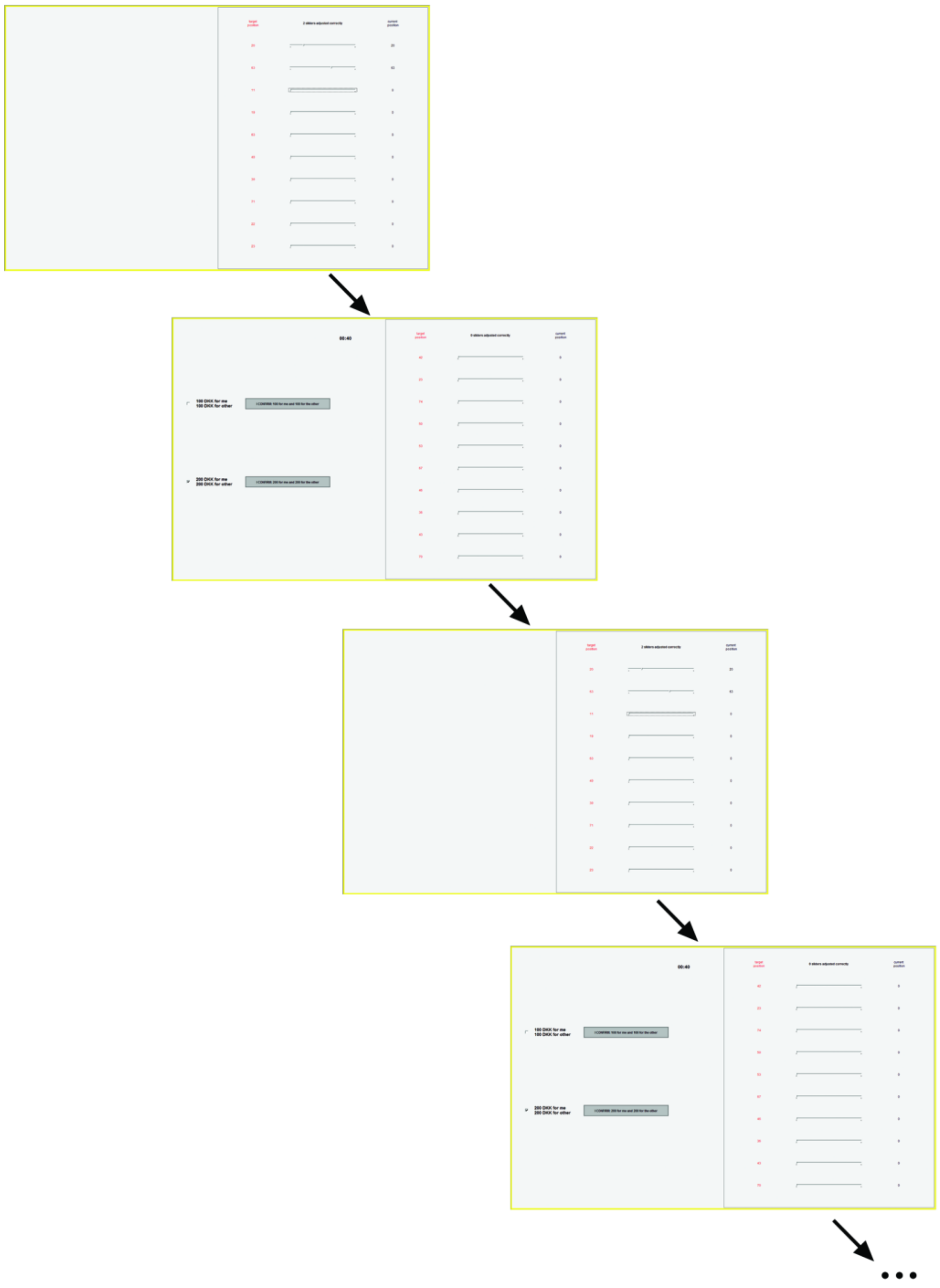

Supplement: S6 File — (PDF) [file pone.0172496.s009.pdf]
