# Supplementary material for: Is there an omission effect in prosocial behavior? A laboratory experiment on passive vs. active generosity
Source: PLoS One. 2017 Mar 1;12(3):e0172496. doi: 10.1371/journal.pone.0172496 (PMC5383002; doi:10.1371/journal.pone.0172496)
Supplement: S7 File — (PDF) [file pone.0172496.s010.pdf]

SUPPORTING INFORMATION S7

Experimental instructions for Experiment 2

This HIT is part of a research project. First, we present to you a scanned text paragraph. You are asked to enter the paragraph word for word into a text box. Make sure to enter the text exactly as it appears in the scanned image. Disregard any line breaks. You can see an example of such a text below. You must complete the paragraph to have your work accepted.

[transcription task]

You and another MTurk worker are matched in this HIT. Note that the other worker is an actual worker and not virtual. Each of you receives a fixed payment of $0.50, but in the following task you can earn an additional bonus.
After you have completed this HIT, you or the other worker that you have been matched with will be chosen at random and both of you will be paid a bonus according to that worker's decisions.
Note that your bonus will be paid through MTurk on top of the fixed payment that you receive. We will make sure to pay the bonuses as soon as the work is completed or the HIT expires.
Press OK to proceed.

No-default treatment

You can select one of two allocations of bonus payments for you ("You") and the other worker ("Other") in this task.
On the next screen, a timer will count down 40 seconds and you will proceed automatically after these 40 seconds. During this time you can select one option. Note that only completed tasks are paid.
The other worker will receive a message that states the details of the choice situation and which bonus payment you selected. You will both be paid accordingly.
Press OK to start the timer.

Please select the allocation you prefer. Note that only completed tasks are paid.
- ❑ You: $1.05, Other: $0.05
- ❑ You: $0.70, Other: $0.70

Commission treatment

You and the other worker receive the following bonus payments for this task:
You: $1.05, Other: $0.05
On the next screen, a timer will count down 40 seconds and you will proceed automatically after these 40 seconds. During this time you can either confirm the bonus payment above or select an alternative option. Note that only completed tasks are paid.

The other worker will receive a message that states the details of the choice situation and which bonus payment you selected. You will both be paid accordingly.
Press OK to start the timer.

Please select the allocation you prefer. Note that only completed tasks are paid.

- ❑ You: $1.05, Other: $0.05
- ❑ You: $0.70, Other: $0.70

Omission treatment

You and the other worker receive the following bonus payments for this task:
You: $1.05, Other: $0.05
On the next screen, a timer will count down 40 seconds and you will proceed automatically after these 40 seconds. During this time you can either confirm the bonus payment above or select an alternative option.
If no choice is made within 40 seconds, we will automatically transfer $1.05 to you and $0.05 to the other worker as bonus payments for this task.
The other worker will receive a message that states the details of the choice situation and which bonus payment you selected, or whether the timer ran down before a choice was made. You will both be paid accordingly.
Press OK to start the timer.

Please select the allocation you prefer. If no choice is made within 40 seconds, we will automatically transfer $1.05 to you and $0.05 to the other worker as bonus payments for this task.

- ❑ You: $1.05, Other: $0.05
- ❑ You: $0.70, Other: $0.70
